# Empathic Concern Is Part of a More General Communal Emotion

**DOI:** 10.3389/fpsyg.2017.00723

**Published:** 2017-05-10

**Authors:** Janis H. Zickfeld, Thomas W. Schubert, Beate Seibt, Alan P. Fiske

**Affiliations:** ^1^Department of Psychology, University of OsloOslo, Norway; ^2^Instituto Universitário de Lisboa (ISCTE-IUL)Lisboa, Portugal; ^3^Department of Anthropology, University of California, Los Angeles, Los AngelesCA, USA

**Keywords:** empathy, empathic concern, sympathy, being moved, kama muta, communal sharing

## Abstract

Seeing someone in need may evoke a particular kind of closeness that has been conceptualized as *sympathy* or *empathic concern* (which is distinct from other *empathy* constructs). In other contexts, when people suddenly feel *close* to others, or observe others suddenly feeling closer to each other, this sudden closeness tends to evoke an emotion often labeled in vernacular English as *being moved, touched*, or *heart-warming* feelings. Recent theory and empirical work indicates that this is a distinct emotion; the construct is named *kama muta*. Is empathic concern for people in need simply an expression of the much broader tendency to respond with kama muta to all kinds of situations that afford closeness, such as reunions, kindness, and expressions of love? Across 16 studies sampling 2918 participants, we explored whether empathic concern is associated with kama muta. Meta-analyzing the association between ratings of state being moved and trait empathic concern revealed an effect size of, *r*_(3631)_ = 0.35 [95% CI: 0.29, 0.41]. In addition, trait empathic concern was also associated with self-reports of the three sensations that have been shown to be reliably indicative of kama muta: weeping, chills, and bodily feelings of warmth. We conclude that empathic concern might actually be a part of the kama muta construct.

## Introduction

When people see others in need, they often respond with compassion and tender feelings, motivating altruistic helping (Batson et al., 1987). This phenomenon has been investigated in the literature on empathy, and variously called empathy, empathic concern, or sympathy. It is widely assumed that individuals differ in how strongly they show this empathic concern, and scales that measure this inter-individual difference are widely used (e.g., Davis, 1980).

Interestingly, empathic concern is often conceptualized as an emotion, but the wider emotion literature mostly ignores empathic concern. Recently, however, the emotion typically labeled in English as *being moved and touched* has received increased attention across philosophy (Cova and Deonna, 2014), aesthetics (Menninghaus et al., 2015), and social psychology (Seibt et al., 2017). In our own work, we have conceptualized the emotion that is often labeled with these English lexemes under the new term *kama muta* (the Sanskrit name of the feeling). We use the term kama muta to emphasize that it is an emotion experienced across many contexts and cultures, with many names—and in some languages, no name (Fiske et al., 2016). In the present paper, we argue that empathic concern can be understood as a special case of kama muta that occurs in response to a specific social situation, namely when perceiving a person (or animal or other agent) in need. To test this idea, we explored the relation between kama muta (measured through labels and reported physiological sensations) and trait empathic concern [measured through Davis’ (1980) Interpersonal Reactivity Index, or IRI] across a meta-analysis of 16 studies with 2918 participants. We thereby link the literature on empathy to the literature on social relations and emotions.

The last decades have seen diverse conceptualizations of *empathy*: Cuff et al. (2016) counted 43 different conceptualizations of it. This shows that concepts of empathy are important to many theorists in the fields of social psychology, personality psychology, affect, and cognition, but they do not agree on what it is. This disagreement means that results from different lines of research cannot be compared, much less build on each other.

One way to make sense of this complexity is to sort different conceptualizations of empathy. As Cuff et al. (2016) point out, many researchers distinguish between affective and cognitive components of empathy (e.g., Davis, 1983a; Baron-Cohen and Wheelwright, 2004; de Waal, 2008). This is certainly a useful way to understand differences and make progress. For instance, a review suggested that cognitive components, such as theory of mind, involve activation of different neural processes than affective components (Singer, 2006).

However, we focus here on another distinction also proposed by Cuff et al. (2016), namely the congruency of the affective reaction: Does empathy mean to feel what we perceive (or assume) other people feeling? In other words, are we empathic when we copy others’ emotions in a congruent way? Several theories consider empathy in this way as a *congruent* (Eisenberg and Fabes, 1990) emotional response to another’s actual or imagined affective state, or as experiencing *similar* feelings (Chismar, 1988). In contrast to that, others argue that empathy implies to feel an incongruent but *appropriate* feeling (Baron-Cohen and Wheelwright, 2004). Interestingly, authors arguing for empathy as including incongruent feelings actually specify what feelings they judge to be appropriate. Responding to someone’s loss with *schadenfreude* would not be considered *empathy* in their terms. Similarly, Cuff et al. state: “Naturally, cases of extreme incongruency, such as feeling anger as a result of mistaking sadness for anger in the target, will represent a failure of empathy”. (p. 148). Instead, the appropriate incongruent but empathic reaction is to feel sympathy or compassion (Hein and Singer, 2008). In this framework, what counts as empathy depends on cultural values for *appropriateness*.

The literature uses various terms for this appropriate incongruent reaction that is considered empathic: *empathic feelings* (Batson et al., 1981), *empathic concern* (Batson et al., 1987) or *sympathy* (Coke et al., 1978; Davis, 1983a). For instance, sympathy has been defined as an affective response to another individual’s emotional state that consists of feelings of concern (Chismar, 1988; Batson, 1990; de Waal, 2008) or sometimes sorrow for the other individual (Eisenberg and Fabes, 1990). The implication of *concern* and *sorrow* is that for the perceiver to feel sympathy, the other must be suffering some sort of distress or misfortune. One would not feel concern or sorrow for someone who just won an award or accomplished a great achievement, or even someone happily walking down the street. And indeed, Davis (1980) and Batson (1990) specified that for the perceiver to feel sympathy or empathic concern, the perceived individual has to be in need or unfortunate. Along with affect, sympathy is sometimes said to have both cognitive and motivational components – understanding the other’s distress *and* desiring to alleviate it (Baron-Cohen and Wheelwright, 2004). Other researchers treat the motivation as a *result of* sympathy, but not a part of it (e.g., Batson et al., 1981; Chismar, 1988). Sympathy has variously been described as the affective part of empathy (e.g., Davis, 1983a; Baron-Cohen and Wheelwright, 2004) or as being caused by empathy (Chismar, 1988; Eisenberg and Fabes, 1990; Singer, 2006). In addition, a large body of literature has investigated the effect of sympathy or empathic concern on altruistic motivated behavior, especially helping (Batson, 2010).

Despite the different labels, definitions, and conceptualizations, it is clear that the incongruent appropriate empathic reaction is supposed to occur with respect to others who are in *need*. This was perhaps most clearly pointed out by Batson et al. (1987), who used the term *empathic concern* for it.

We argue that empathic concern can be best understood as a special case, evoked by a particular situation (another’s need) of an emotion that has been mentioned by emotion scholars since Darwin (1872) and James (1890) but until recently has received little sustained theoretical or empirical attention. This emotion is also evoked by many social situations that do not involve anyone being in need—but the social situations that evoke the emotion can be precisely conceptualized, and have been empirically tested. Our goal in the current work is to explore whether this emotion, which we call kama muta, encompasses the specific phenomenon that has previously been labeled empathic concern, sympathy, compassion, etc.

To understand what the emotion behind such labels as empathic concern actually is, we can look at how it is measured. Coke et al. (1978) and Batson et al. (1987) assessed their construct of the state of empathic concern with six self-report affective items: *sympathetic*, *compassionate*, *tender*, *softhearted*, *warm*, and *moved*. Similarly, to measure the general tendency to have such reactions, the IRI subscale on trait empathic concern Davis (1980) includes an item with the statement: “I am often quite touched by things that I see happen”. This resonates with Baron-Cohen and Baron-Cohen and Wheelwright’s (2004, p. 165) note that “if you walk past a homeless person in winter and you are ‘moved’ or ‘touched’ (both interesting metaphors) to want to help them, this would count as sympathy”.

Clearly, these authors rely on their subjects’ and readers’ understanding of the vernacular labels of feeling *moved*, *touched*, and having *warm feelings*. Other scholars of emotion have recognized and discussed these lexemes’ reference to a distinct emotion, mostly termed *being moved*, starting with Darwin (1872) and James (1890), and later Panksepp (1995), and Tan and Frijda (1999). The literature can be summarized as arguing that this emotion has four aspects, namely (1) the typical sensations, (2) positive valence, (3) a specific kind of motivation, and (4) a specific kind of appraisal. Let us look at each in turn.

(1) It’s typically recognized that the feeling is accompanied by a lachrymal reaction such as moist eyes or weeping, which later scholars have also noted (Miceli and Castelfranchi, 2003; Cova and Deonna, 2014; Vingerhoets and Bylsma, 2016; Seibt et al., 2017). Further theorization and empirical studies have linked the feeling that participants label being moved with experiencing warmth in the body, especially in the center of the chest near the heart (Tan and Frijda, 1999; Schnall et al., 2010; Cova and Deonna, 2014; Zickfeld, 2015; Schubert et al. 2016, Seibt et al., unpublished). Goosebumps or chills have been identified as a third physiological symptom generally occurring with being moved (Benedek and Kaernbach, 2011; Strick et al., 2015; Wassiliwizky et al., 2015, 2017; Schubert et al., 2016; Seibt et al., 2017).
(2) Although often accompanied by moist eyes or crying, the state usually has been posited and found to be experienced as generally positively valenced (Cova and Deonna, 2014; Menninghaus et al., 2015; Seibt et al., unpublished). Some studies have shown that negative affects can occur at the same time and have characterized being moved as a mixed emotion (Hanich et al., 2014; Menninghaus et al., 2015). (We will return to this in the Discussion).
(3) In general it has been theorized that feeling *moved* motivates individuals to act communally, share and care (Menninghaus et al., 2015; Fiske et al., 2016). One study found an increase of identification with humanity after watching a moving video in contrast to control videos (Oliver et al., 2015). Further evidence suggested that feeling moved mediates the effect of emotional videos on altruistic behavior toward another individuals (Schnall et al., 2010). (Although the last two studies were conducted with reference to the *elevation* construct, the researchers assessed elevation by asking informants to rate their feelings of being *moved* and *warmth*, among other items, and focused in their analyses on these two items).
(4) Various theories have been proposed about the elicitors of being *moved*. Cova and Deonna (2014) argued that feelings of being moved occur when *positive core values arise and transform from negative values;* such values must have an identifiable cause (a formal object). The authors defined core values as values that have significance for a moral community (Tetlock et al., 2000) and presented evidence from thought experiments utilizing *être ému* (being moved) as their main indicator. Introducing a related construct, *elevation*, Haidt and colleagues described affective states that are elicited by observation of situations high in purity and moral virtue (Silvers and Haidt, 2008; Algoe and Haidt, 2009). They equated elevation with being moved (Haidt, 2003) and have also utilized *being moved* as an indicator item to measure elevation (e.g., Schnall et al., 2010; Thomson and Siegel, 2013). Studies of elevation have mostly focused on its consequences and functions (Thomson and Siegel, 2016). A further theorization is provided by Menninghaus et al. (2015), who argued that being moved is evoked by, inter alia, significant relationship events that are high in compatibility with pro-social norms.

To provide a comprehensive model of this emotion, we introduced the kama muta model (Fiske et al., 2016). Vernacular language labels are imprecise and their semantics differ considerably over time and across cultures; we therefore label the emotion kama muta (Sanskrit for moved by love). The model is based on Relational Models Theory (RMT, Fiske, 2004), and especially on the relational model of communal sharing described in that theory. Communal sharing relationships are generally characterized by a sense of social equivalence that is indexed by common or shared essences such as group membership or kinship. It is characterized by helping according to need and ability (Fiske, 2004).

The kama muta model integrates the previously listed ideas and findings regarding sensations, motivations, and valence, but posits that kama muta occurs when communal sharing relationships suddenly intensify (Fiske et al., 2016; Schubert et al., 2016). For example, singing the national anthem might evoke kama muta because a common essence and identity are highlighted. All relational models are based on two complementary aspects: evolved templates and culturally specific implementations. The same is true for social emotions like kama muta: The sudden intensification of a communal sharing relation describes the evolved universal template of kama muta, while the feeling is labeled, interpreted, and evaluated differently depending on its cultural implementation (e.g., *being moved; être ému* in French*, å være rørt* in Norwegian*, gerührt und bewegt sein* in German, *gan dong* in Chinese, etc.; Seibt et al., unpublished).

The main difference between the kama muta model and previous *being moved* constructs lies in its reduced focus on linguistic labels, its accounting for cultural variation, and the linking of its appraisal pattern to a basic social relational model: communal sharing. It also specifies that kama muta occurs when a communal sharing relationship suddenly intensifies, but not when other virtues or values are realized. Otherwise, the predictions of the kama muta model resemble the predictions of older models regarding valence, physiology, feeling or motivation.

Is there an overlap between the kama muta state and what has been called *empathic concern*? There are clear similarities in labeling the feeling state (moving, touching, warm), sensations and physiological symptoms (warmth), and motivation (altruistic helping). Regarding appraisals, the kama muta model proposes that sudden intensifications of communal sharing (or other contrasts of communal sharing against a backdrop of its lack) cause the emotion. The question is how situations in which one perceives another in need, and feels empathic concern, constitute an intensified communal sharing relation, and thus fit the appraisal. Our working hypothesis is that the display of need and its appeal to share communally constitutes this intensification. (We will return to this hypothesis and the specificity of the kama muta model in the Discussion, because those are not tested by the data we present below).

In sum, we propose that what is typically described as *empathic concern, sympathy*, and *compassion* in the empathy literature are instances of *kama muta*, an emotion that regulates communal sharing relations. Our approach is congruent with that of previous authors who have also linked feeling moved to the concept of empathy (Yarrow et al., 1976; Tokaji, 2003; Menninghaus et al., 2015; Bartsch et al., 2016).

A few studies have already provided data on this question. One study reported a positive correlation [*r*_(132)_ = 0.35] between trait empathy ratings as measured by the Empathy Quotient (EQ; Baron-Cohen and Wheelwright, 2004) and the frequency of recalled experiences of *being moved* (*être ému*; Cullman, 2015). Participants were instructed to write about experiences that made them feel *moved*; these were coded for intensity and frequency. The results suggest that being more trait-empathic is associated with more frequently feeling *moved*, while there was no association with recalled intensity of the experiences. It should be noted that the EQ does not differentiate between affective and cognitive empathy, or distinguish empathy from sympathy in response to need. Hence, it is not clear whether results from this study were directly related to empathic concern.

In contrast to the EQ, the IRI (Davis, 1980) does differentiate four aspects: Empathic concern as introduced above, perspective taking (the cognitive component of empathy), personal distress, and *fantasy*. The personal distress scale assesses one’s own distress in response to negative and challenging situations. The fantasy subscale measures empathy with fictional characters; it is debated whether this is a more cognitive or affective variant of empathy (Jordan et al., 2016).

In one study Davis (1983b) directly tested the relation between his trait empathic concern measurement and the state empathic concern concept of Batson et al. (1981). Participants were instructed to either “objectively” listen to a tape recording of a needy person, or to actively “identify with the feelings and reactions” of the person (p. 173). Davis assessed state empathic concern using a measure that included items on *moved*, and *touched*. He found a positive correlation between trait and state empathic concern, *r*_(156)_ = 0.28, [95% CI: 0.13, 0.42]. In the context of research correlating personality traits with emotional states, this seems to be a relatively strong association (Robins et al., 2009). Of course, the question is whether this finding can be generalized to other situations of perceived need, or how specific this correlation is. Davis also presented a correlation of similar magnitude between trait empathic concern and the state of personal distress while listening to the tape of the needy person. He concluded that the empathic concern subscale of the IRI might just measure a general tendency of emotionality and therefore correlate highly with *any* affective state (cf. Davis, 1983a). In the Davis (1983b) study, state empathic concern was not significantly predicted by trait perspective taking, emphasizing the difference between these constructs.

Similarly, a recent study investigated the relation of empathic concern to *moving sadness* while listening to music, a concept measured with self-reports items such as *moved, intensity, liking*, and *sadness* (Eerola et al., 2016). The authors also reported a positive correlation, *r*_(99)_ = 0.35, [0.17, 0.51], between moving sadness and the Davis’ (1980) trait empathic concern measure. In addition, they also found a positive correlation, *r*_(99)_ = 0.38, [0.20, 0.54], between moving sadness state and the fantasy subscale of the IRI.

We therefore theorize that the state of empathic concern is a specific case of experiencing the emotion kama muta when perceiving others in need. According to our kama muta theory, therefore, a self-reported tendency (trait disposition) to easily and strongly feel empathic concern should predict two things: First, persons who are high on that trait should report being moved and touched in a broad range of situations, including those where perceived need is not the primary aspect of the situation. Second, individuals high in trait empathic concern should report the full spectrum of physical sensations of kama muta in situations in which they perceive communal sharing to intensify suddenly although they do not perceive anyone needy. This second point means that high empathic concern should predict more than just self-reports of vague “feeling of warmth,” a typical but ambiguous measure which participants may interpret metaphorically as interpersonal ‘warmth.’ When observing sudden intensification of communal sharing, high trait-empathic concern should predict participants’ reports of warmth *in their own chests or bodies*, together with moist eyes or tears, and goosebumps or chills.

With respect to the congruent view of empathy, note that our theory connecting trait empathic concern to kama muta posits that kama muta is distinct from other emotions, so trait empathic concern should *not* substantially or consistently predict feeling other emotion states such as happiness, amusement, sadness, or fear. We also argue that in general *only* empathic concern should be related to kama muta, not perspective taking or the other subscales of the IRI.

Hence, we comprehensively test the relation between each of the trait subscales of the IRI and state reports of kama muta, across a variety of situations. Our stimuli vary from short video clips to self-written narratives, but generally differ from classic stimuli in “sympathy” studies (e.g., manipulated neediness, vignettes) in that they do not focus on the needy state.

Based on the conceptualizations and evidence reviewed above, we predict that:

(1) The intensity of feeling *moved* or *touched* (the feeling component of kama muta) is positively related to trait empathic concern, as measured by that subscale of the IRI.
(2) The self-reported intensities of the physical sensations weeping, chills, and warmth (the physiological components of kama muta) are positively related to trait empathic concern.
(3) Trait empathic concern is not consistently related to participants’ reports of affective states other than kama muta.

## Overview of the Current Studies

Our hypotheses were tested in a total of sixteen different studies. The sixteen studies employed various samples and designs, as well as eliciting being moved with various types of stimuli. They were mainly conducted to test other hypotheses, but included measures of IRI scales, and were thus informative for the present question. We meta-analytically combined effects sizes of the different studies to test our main hypotheses.

After analyzing thirteen studies we pre-registered a replication of the main effect, specifying Study 16 based on the effect size obtained until then^1^. The pre-registration was conducted to supplement the collected data and address possible issues concerning study design. In particular, Study 16 differed from all other studies in that it measured the IRI first, before showing any stimulus, in order to rule out the possibility that the reported trait reflects the emotion just experienced in response to our stimuli. Studies 14 and 15 were conducted after the pre-registered replication.

For each study we report how we determined our sample size, all data exclusions (if any), all manipulations, and all measures (see Supplementary Material). We report *all studies* we have conducted (at the point where we began the data analyses) that included both of the main measures of interest (IRI and the items *being moved* or *touched*). In other words, our file drawer is empty. All data files are available at osf.io^2^ except for parts that might identify participants.

All of our protocols were reviewed and approved by the Internal Review Board of the University of Oslo. In all studies participants were explicitly asked to indicate consent by proceeding with the study.

## Meta-Analysis of Sixteen Studies

**Table 1** provides an overview of the studies. A more detailed description of each study is provided in the Supplementary Material. The following methods section will present and aggregate information for all studies.

**Table 1:** Overview of the sixteen studies including population, sample size design, and kind of stimuli.

#	*N*	Cases	Female/Male	Age (*M*, *SD*)	Population	Design	IRI measures	Stimuli	Other
1	80	605	47/33	20–59 (34.01, 9.44)	US American MTurk	Within	All	Narratives	
2	152	456	63/89	18–67 (32.95, 9.85)	US American MTurk	Within	All	Videos	
3	93	246	69/21/3	19–50 (24.12, 5.91)	Norwegian Undergraduates	Within	All	Videos	
4	140		73/67	20–69 (35.58, 12.02)	US American MTurk	Correlational	PT, EC	Videos	
5	115		46/69	19–60 (33.57, 10.69)	US American MTurk	Between	All	Videos	
6	111		75/35/1	19–52 (24.23, 6.26)	Norwegian Undergraduates	Between	PT, EC	Videos	
7	219	438	122/95/2	19–63 (31.67, 10.70)	US American MTurk/Norwegian Normal Adults	Within	EC	Videos	
8	54		25/27/2	20–52 (30.24, 7.67)	US American MTurk	Correlational	All	Self-written	
9	52		19/32/1	21–51 (29.52, 6.50)	US American MTurk	Correlational	All	Self-written	
10	400		198/201/1	19–69 (36.09, 11.70)	US American MTurk	Between	PT, EC, PD	Audio Narratives	
11	305		183/121/1	18–70 (33.82, 9.77)	US American MTurk	Between	PT, EC, PD	Self-written Narratives	
12	220	752	139/75/6	19–69 (31.71, 12.39)	US American MTurk/Norwegian Undergraduates	Within	All	Videos	Seibt et al., 2017
13	237	474	136/98/3	19–69 (30.31, 10.64)	US American MTurk/Norwegian Undergraduates	Within	All	Self-written Narratives	Seibt et al., 2017
14	313		160/153	18–74 (36.62, 11.57)	US American MTurk	Correlational	EC	Videos	
15	138		104/31/3	19–63 (25.04, 8.12)	Norwegian Undergraduates	Correlational	EC	Videos	
16	289	793	161/126/2	19–67 (29.64, 10.04)	US American MTurk/Norwegian Undergraduates	Within	All	Videos	Pre-Registered
									(https://osf.io/xguev/)

## Materials and Methods

### Participants

In total 2918 participants were recruited via different channels for the final sample^3^. Of these, 2087 US American participants were sampled on Amazon MTurk requesting only workers with at least 95% approval rate (Studies 1, 2, 4, 5, 7, 8, 9, 10, 11, 13, 14, and 16); and 742 Norwegian normal adults completed the studies for course credit or voluntarily on social media (Studies 3, 6, 7, 12, 13, 15, and 16; 89 participants are other nationality or unspecified). The final dataset contained 1613 females and 1280 males (25 unspecified) ranging from 18 to 74 years of age (*M* = 32.2, *SD* = 11.0).

### Overview and Design

The present studies were introduced as investigating the relation between emotions and media. Designs differed slightly across studies. Nine of the studies utilized a within design (1 – 3, 7, 12 – 13, 15 – 16) with each participant rating, watching, or reading more than one stimulus, while the rest employed a between-participants approach (5 – 6, 10 – 11), with individuals being randomly allocated to two (6) or three different conditions (5, 10 – 11), or finally, a correlational design (4, 8 – 9, 14). All sixteen studies were conducted online.

### Materials

The sixteen studies employed a wide array of different stimuli types. Eleven of the studies (2 – 7, 12 – 16) utilized video stimuli that were adopted from earlier research (Schubert et al., 2016; Seibt et al., unpublished) and featured a broad spectrum of themes. The studies relied mostly on videos judged as moving in earlier findings (2 – 6, 12 – 16; Schubert et al., 2016), but also included other videos intended to evoke other emotions such as fear, happiness, or sadness (12); amusement, awe, or sadness (16); or videos showing cute versus non-cute animals (7, 15). One study (1) presented participants with narratives that were produced by participants in a pilot study with the instructions to write a story that would make other people “moved or touched”. Two studies asked participants to think of and very briefly write about different their own personal experiences that were moving (11, 13), sad (13), neutral, or humorous (11). Another study (10) employed audio narratives that were, respectively, intended to induce feelings of being moved, amusement, or a neutral affect. Two approaches asked participants to remember and report episodes including positive tears on Valentine’s day (8), or episodes with increased communality on Valentines day (9). The present data have not been reported previously, except for some measures in two studies (12, 13) in which other analyses including some of the same variables were previously reported in Seibt et al. (2017).

Upon watching, listening to, or reading the stimuli, or reporting an experience, participants were always presented with two questions assessing their feelings of being *moved* or *touched* with regard to the materials. These two items were rated on either 5-point (2, 5, 6, 12) or 7-point scales (1, 3, 4, 7, 8, 9, 10, 11, 13 – 16) anchored at *not at all* and *very much*. (The instructions differed slightly, depending on whether participants were asked about videos, narratives, or self-reported stories). In studies 10 – 13, and 16, participants were also presented with items asking whether they were *amused* (10 – 11, 16), *sad* (12, 13, 16), *happy* (10 – 13), *awed* (16), or *anxious* (12). Items were completed on either 5-point (12) or 7-point scales ranging from *not at all* to *very much*. Afterward participants were asked about a number of physiological sensations that were rated on either 3-point (2, 5) 5-point (1, 6, 10, 11, 12, 13) or 7-point scales (3, 4, 7, 8, 9, 14, 15, 16) anchored at *not at all* and *very much* (1, 6, 10, 11, 12, 13) or in some cases the high anchor was *clearly* (2, 5) or *a lot* (3, 4, 7, 8, 9, 14, 15, 16). These items differed minimally across studies, some studies employed three items asking, respectively, about tears/moist eyes, warm chest, and chills/goosebumps; other studies employed two items targeting tears and moist eyes separately (7, 10, 11, 13, 14, 15, 16), or two items asking about chills and goosebumps separately (3, 4, 7, 8, 9, 14, 15, 16).

Finally, in every study participants were presented with the IRI (at the end in every study but 16). Participants completed items on a 5-point scale anchored as *does not describe me well* and *describes me very well*. In eight studies participants responded to all four subscales on perspective taking, empathic concern, personal distress, and fantasy (1 – 3, 5, 8 – 9, 12 – 13, 16), while two studies did not include the fantasy scale (10, 11), two studies included only the perspective taking and empathic concern scales (4, 6), and finally three studies used only the empathic concern scale (7, 14 – 15). **Table 2** presents the reliabilities of the four subscales in each study in which they were used. In order to address possible presentation-order effects, the pre-registered replication (16) presented the IRI at the beginning before the participants watched any videos.

**Table 2:** Overview of regression models with the being moved index regressed on perspective taking (PT), empathic concern (EC), personal distress (PD), and fantasy (FT).

#	Analysis model	IRI Scale	α	*B* [95% CI]	df	*t*	*p*
1	Multilevel model	PT	0.84	-0.31 [-0.70, 0.08]	75	-1.57	0.121
		EC^∗∗^	0.85	1.24 [0.85, 1.63]	78	6.27	<0.001
		PD	0.88	-0.11 [-0.41, 0.18]	78	-0.77	0.441
		FT	0.80	0.03 [-0.29, 0.36]	77	0.19	0.853
2	Multilevel model	PT	0.88	-0.20 [-0.45, 0.06]	147	-1.52	0.134
		EC^∗^	0.89	0.40 [0.16, 0.64]	147	3.26	0.001
		PD	0.86	-0.03 [-0.18, 0.12]	147	-0.40	0.691
		FT	0.84	0.16 [-0.01, 0.33]	147	1.82	0.070
3	Multilevel model	PT	0.74	-0.09 [-0.54, 0.36]	87	-0.41	0.687
		EC	0.72	0.45 [-0.05, 0.95]	82	1.78	0.079
		PD	0.77	0.22 [-0.11, 0.54]	88	1.30	0.197
		FT	0.82	0.44 [0.07, 0.81]	87	2.35	0.021
4	Linear regression	PT	0.90	-0.03 [-0.42, 0.36]	137	-0.16	0.872
		EC^∗∗^	0.90	0.97 [0.60, 1.34]	137	5.12	<0.001
5	Linear regression	PT	0.90	0.23 [-0.12, 0.57]	110	1.30	0.198
		EC	0.88	0.22 [-0.10, 0.55]	110	1.33	0.186
		PD	0.84	0.02 [-0.23, 0.28]	110	0.16	0.872
		FT	0.79	-0.06 [-0.36, 0.24]	110	-0.40	0.690
6	Linear regression	PT	0.77	-0.10 [-0.50, 0.30]	108	-0.50	0.622
		EC^∗∗^	0.75	0.91 [0.52, 1.30]	108	4.51	<0.001
7	Linear regression	EC^∗^	0.88	0.53 [0.28, 0.78]	216	4.16	<0.001
8	Linear regression	PT	0.84	0.33 [-0.54, 1.20]	49	0.74	0.462
		EC	0.90	-0.18 [-0.84, 0.48]	49	-0.54	0.594
		PD	0.92	0.15 [-0.19, 0.50]	49	0.87	0.388
		FT^∗^	0.76	1.09 [0.30, 1.88]	49	2.72	0.009
9	Linear regression	PT	0.83	0.36 [-0.40, 1.13]	47	0.93	0.359
		EC	0.86	-0.07 [-0.71, 0.57]	47	-0.21	0.835
		PD	0.84	-0.20 [-0.74, 0.33]	47	-0.75	0.456
		FT	0.84	-0.08 [-0.62, 0.47]	47	-0.27	0.786
10	Linear regression	PT	0.88	0.001 [-0.37, 0.37]	133	0.01	0.994
		EC^∗∗^	0.89	1.06 [0.77, 1.34]	133	7.20	<0.001
		PD	0.89	-0.06 [-0.28, 0.16]	133	-0.50	0.618
11	Linear regression	PT	0.86	0.26 [-0.43, 0.95]	85	0.73	0.466
		EC^∗^	0.88	0.69 [0.21, 1.18]	85	2.81	0.006
		PD	0.87	0.13 [-0.27, 0.52]	85	0.64	0.526
12	Linear regression	PT	0.84	-0.09 [-0.36, 0.18]	198	-0.64	0.523
		EC^∗∗^	0.87	0.79 [0.50, 1.07]	198	5.45	<0.001
		PD	0.84	-0.08 [-0.28, 0.12]	198	-0.82	0.411
		FT	0.79	0.04 [-0.22, 0.30]	198	0.28	0.777
13	Linear regression	PT	0.82	-0.08 [-0.42, 0.26]	231	-0.47	0.642
		EC	0.83	0.42 [0.08, 0.75]	231	2.45	0.015
		PD	0.87	0.04 [-0.20, 0.27]	231	0.31	0.754
		FT	0.82	0.24 [-0.02, 0.50]	231	1.84	0.067
14	Linear regression	EC^∗∗^	0.82	0.91 [0.70, 1.11]	311	8.50	<0.001
15	Linear regression	EC^∗^	0.76	0.55 [0.13, 0.98]	133	2.55	0.012
16	Linear regression	PT	0.83	-0.10 [-0.39, 0.19]	273	-0.67	0.507
		EC^∗∗^	0.84	0.76 [0.45, 1.06]	273	4.87	<0.001
		PD	0.86	-0.04 [-0.27, 0.19]	273	-0.34	0.732
		FT	0.82	0.32 [0.06, 0.57]	273	2.44	0.015

*95% confidence intervals are not Bonferroni corrected. ^∗^*p* < 0.05/number of predictors in model, ^∗∗^*p* < .001*.

After completing ratings on the stimuli, participants in all studies were asked to provide demographic information, and finally debriefed.

## Results

Some of the studies (10 – 13, 16) presented participants with conditions that were chosen to induce different emotional states. For the analyses of these studies we retained only the cases that involved stimuli or events in the being moved condition. Therefore, most analyses reported here from studies 10 – 13 and 16 do not employ the total sample of participants (see **Table 3**). We tested all studies with the total sample as well and effect sizes were minimally different, generally a bit smaller. In addition, the studies employed a number of different scales. Because the main analyses present standardized estimates we did not transform scales into a unified metric.

**Table 3:** Overview of descriptives and reliabilities of main measures for each study separately.

Study #	*N* (cases)	Measure						
		Feeling moved		Tears		Warmth	Chills	
		α	*M* (*SD*)	α	*M* (*SD*)	*M* (*SD*)	α	*M* (*SD*)
1	80 (605)	0.94	4.50 (1.94)		1.64 (1.11)	1.93 (1.34)		1.56 (1.07)
2	152 (456)	0.97	3.54 (1.23)		1.81 (0.86)	1.81 (0.80)		1.56 (0.79)
3	91 (243)	0.94	4.51 (2.02)		2.49 (1.98)	4.25 (1.87)	0.90	2.65 (1.82)
4	140	0.91	5.26 (1.68)		2.93 (2.17)	4.43 (2.00)	0.91	3.01 (2.09)
5	115	0.96	3.89 (1.17)		1.83 (0.87)	1.98 (0.83)		1.56 (0.79)
6	111	0.92	3.79 (1.25)		1.50 (0.97)	2.39 (1.16)		1.59 (0.95)
7	218	0.93	2.67 (1.79)	0.79	1.36 (0.95)	2.89 (1.96)	0.90	1.30 (0.87)
8	54	0.74	5.74 (1.48)		5.59 (1.25)	5.30 (1.71)	0.74	3.72 (1.74)
9	52	0.75	4.97 (1.44)		3.10 (2.14)	4.73 (1.88)	0.85	3.48 (1.73)
10	137	0.96	5.73 (1.53)	0.91	2.01 (1.39)	2.76 (1.48)		2.09 (1.38)
11	89	0.92	5.44 (1.77)	0.88	2.10 (1.42)	2.98 (1.45)		2.42 (1.41)
12	204	0.91	5.93 (1.27)		3.22 (1.61)	3.24 (1.43)		2.73 (1.62)
13	236	0.88	5.72 (1.58)	0.73	3.78 (1.09)	3.81 (1.16)		3.02 (1.48)
14	313	0.91	4.33 (1.75)	0.90	1.99 (2.09)	2.89 (2.07)	0.85	1.49 (1.80)
15	138	0.86	2.66 (1.70)	0.86	1.28 (0.95)	3.37 (1.94)	0.83	1.36 (.97)
16	278	0.87	5.31 (1.64)	0.89	3.32 (2.15)	4.03 (2.00)	0.82	2.87 (1.93)

*Number of cases is given for the final number of participants employed in the main meta-analysis of the being moved index and empathic concern. Note that these differ from **Table 1** because of possible missing values and exclusion of conditions that were intended to evoke non-moving feelings. Descriptives are presented for within study comparisons. Between study comparisons should be avoided because of different scales*.

### Index of Feeling Moved

For all studies, ratings on *being moved* and *being touched* were combined into a composite index, which we label here *feeling moved* (see Seibt et al., 2017). Reliabilities assessed by Cronbach’s alpha ranged from 0.74 to 0.96, which are sufficient. The main dependent variable in all analyses was this feeling moved index.

### Feeling Moved and Empathic Concern

In order to test the relation between feeling moved and trait empathic concern, we regressed feeling moved on each of the four subscales of the IRI that were included in the respective studies, without including their interactions. For some studies it was only possible to regress feeling moved on three (studies 10 and11), two (4 and 6), or one subscale (7, 14, 15). Because the studies utilized different designs we employed a range of different statistical models (see **Table 2** for an overview). Studies 1 – 3 were analyzed utilizing hierarchical linear models because each participant provided more than one rating, while studies 4 – 16 were analyzed by linear regression. For the multilevel models, intercepts were always allowed to vary accordingly to participant. We applied a Bonferroni correction to all studies utilizing two or more predictors. Empathic concern had an effect in eleven out of sixteen cases, using the criterion of a 95% Bonferroni-corrected confidence interval. Using this criterion, the Fantasy subscale had an effect in only one study, while Personal Distress and Perspective Taking had no effect in any study (**Table 2**).

**FIGURE 1:**
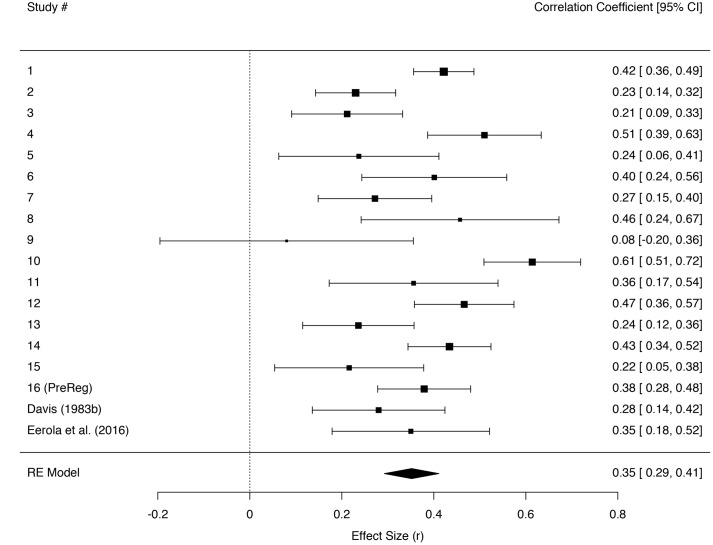
**Forest plot of effect sizes (*r*) for regressing being moved on the subscale of empathic concern individually across the sixteen different studies and previous evidence by Davis (1983b) and Eerola et al. (2016) in a random effects meta-analysis**. Error bars indicate 95% confidence intervals. Heterogeneity tests: *Q*(17) = 69.04, *p* < 0.001, *I^2^* = 75.43 [54.33, 89.59].

In order to test whether the effect of empathic concern is independent of the other empathy subscales, a model with only the empathic concern subscale^4^ as predictor was computed for each study. Unstandardized *Bs* were standardized and then treated as effect sizes *r* (cf. Bowman, 2012). The effect sizes were combined in a meta-analysis utilizing the R software *metafor* package (Viechtbauer, 2010). A random effects model was specified with a restricted maximum-likelihood estimation (REML). We added two effect sizes from previous research reported by Davis (1983b) and Eerola et al. (2016) that tested empathic concern and states labeled as *touched* or *moved*. **Figure 1** presents an overview of the effect sizes and the random effects estimate, together with heterogeneity tests. Seventeen of the eighteen effects were significant according to a 95% confidence interval criterion. Relying on the four published effect sizes, the overall estimate of random effects of trait empathic concern on intensity of the state of feeling moved by our stimuli is *r*_(3631)_ = 0.35 [0.29, 0.41]^5^.

A second meta-analysis was fitted in order to explore possible effects of nationality. Sample effect sizes were divided into US and Norwegian and analyzed using a mixed-effects model and a REML estimation. Results suggested that US effect sizes were on average higher than Norwegian ones (*b* = 0.08, *SE* = 0.07), but this difference was not significant, *z* = 1.22, *p* = 0.22^6^. Finally, we also explored possible gender effects by using a mixed-effects model and a REML estimation. Effect sizes from female participants were on average higher than those from male ones (*b* = 0.04, *SE* = 05), but this difference was not significant either, *z* = 0.70, *p* = 0.48.

### Sensations and Empathic Concern^7^

**FIGURE 2:**
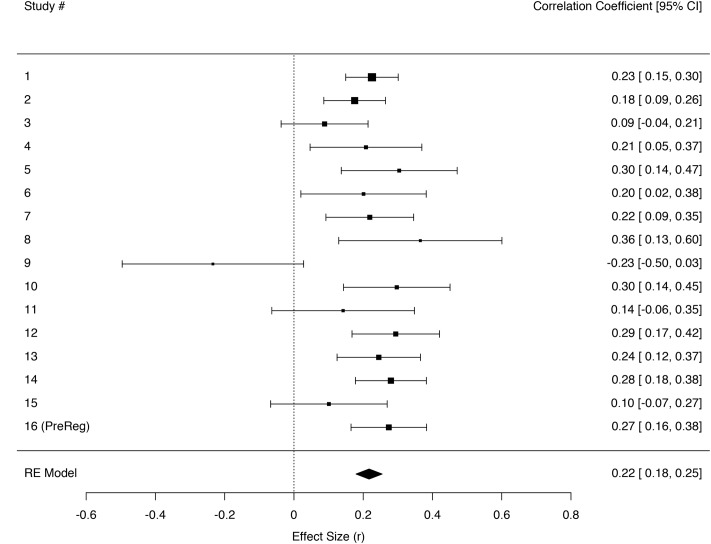
**Forest plot of effect sizes (*r*) for regressing being moved on the tears measure individually across the sixteen different studies in a random effects meta-analysis**. Error bars indicate 95% confidence intervals. Heterogeneity tests: *Q*(15) = 26.40, *p* = 0.034, *I^2^* = 22.39 [0.00, 87.83].

**FIGURE 3:**
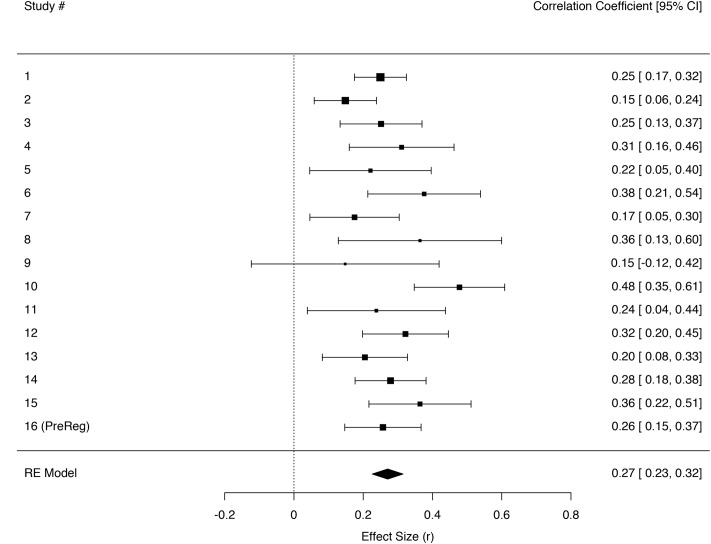
**Forest plot of effect sizes (*r*) for regressing being moved on the warmth item individually across the sixteen different studies in a random effects meta-analysis**. Error bars indicate 95% confidence intervals. Heterogeneity tests: *Q*(15) = 26.15, *p* = 0.037, *I^2^* = 44.85 [0.00, 76.54].

**FIGURE 4:**
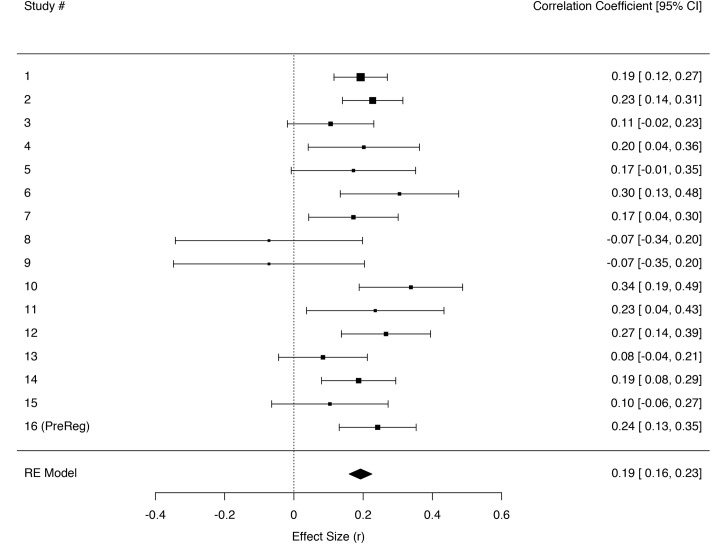
**Forest plot of effect sizes (*r*) for regressing being moved on the chills item individually across the sixteen different studies in a random effects meta-analysis**. Error bars indicate 95% confidence intervals. Heterogeneity tests: *Q*(15) = 21.16, *p* = 0.13, *I^2^* = 2.15 [0.00, 81.60].

Three independent meta-analyses were fitted in order to test the relation between trait empathic concern and the sensations tears, chills, and warmth. For some studies measures for tears and chills were created by combining two separate items on tears and moist eyes or chills and goosebumps, respectively (see **Table 3** for Study overview and reliabilities). A random effects model using REML gave an effect size estimate of *r*_(3363)_ = 0.27 [0.23, 0.32] for participants’ feelings of warmth in their own bodies; *r*_(3366)_ = 0.22 [0.18, 0.25] for tears; and *r*_(3360)_ = 0.19 [0.16, 0.23] for chills (**Figures 2**–**4**).

### Empathic Concern and Other Affective States

In the next step, we explored whether empathic concern is uniquely related to being moved or also predicts other affective states. For these models we only included studies presenting stimuli intended to evoke other emotional states in addition to being moved. (We collected answers on other emotions across all studies, but most of them relied on video or other stimuli selected to evoke kama muta, therefore offering little variance on other affective responses). Studies 10 – 13 and 16 included different stimuli intended to evoke different emotions. In Studies 10, 11, and 16 amusement was regressed on empathic concern. In Study 12 happiness, sadness, and fear were regressed on empathic concern, each in a separate model. For Study 13 and 16, sadness was regressed on empathic concern. Finally for Study 16, awe was regressed on empathic concern. All studies utilized a linear regression procedure.

**Table 4** presents an overview of the results. Based on a 95% inference criterion, the states happiness and sadness (Study 12, 13, and 16) were significantly predicted by trait empathic concern. In one study (11), empathic concern predicted amusement, but in two other studies (10, 16) it did not. Empathic concern did not predict fear in the study (12) that presented stimuli meant to evoke fear (and that actually evoked fear in that condition). Empathic concern did not predict awe in the study (13) that presented stimuli meant to evoke awe (and that actually evoked awe in that condition).

**Table 4:** Overview of regression models with different affective response items regressed on the subscale of empathic concern.

#	Analysis model	Affective response	*β* [95% CI]	df	*t*	*p*
10	Linear regression	Amusement	-0.14 [-0.67, 0.07]	128	-1.57	0.119
11	Linear regression	Amusement	0.27 [0.09, 0.44]	106	2.94	0.004
12	Linear regression	Happiness	0.34 [-0.01, 0.14]	175	4.80	<0.001
		Sadness	0.44 [0.09, 0.23]	172	6.38	<0.001
		Fear	-0.13 [-0.02, 0.13]	174	-1.77	0.078
13	Linear regression	Sadness	0.18 [0.05, 0.30]	235	2.76	0.006
16	Linear regression	Amusement	0.06 [-0.05, 0.37]	187	0.75	0.454
		Awe	0.02 [-0.10, 0.38]	130	0.27	0.790
		Sadness	0.34 [0.19, 0.57]	195	5.01	<0.001

*Each emotion item is regressed in a separate model*.

## General Discussion

We compared ratings of kama muta and trait empathic concern, utilizing a broad variety of stimuli across 16 different studies sampling 2918 participants. Across most studies there was a positive relationship between the intensity of the feeling component of kama muta as measured by *being moved* and *being touched* and trait empathic concern as assessed by the IRI scale. Confirming our second hypothesis, self-reported sensations of kama muta, namely weeping, chills, and bodily warmth, were also positively associated with trait empathic concern. The third hypothesis was confirmed for three emotions but not for sadness and happiness. While affective state ratings of fear, amusement, and awe were not consistently related to ratings of trait empathic concern, states of sadness exhibited a relationship with trait empathic concern for all tested studies. We also found a relationship with state happiness in one study.

Taking the total 18 studies into account, four published (Davis, 1983b; Eerola et al., 2016; Seibt et al., 2017), thirteen unpublished, and one pre-registration, the overall effect size between state ratings of feeling moved and trait empathic concern was *r* = 0.35. This is a large effect relative to most research comparing the intensity of an affective state with a personality trait, and relative to many other kinds of research in personality and social psychology (Richard et al., 2003; Roberts et al., 2007; Robins et al., 2009). Based on the evidence presented it can be inferred that the two concepts, kama muta and empathic concern, are highly associated with each other, lending support to our hypothesis that instances of empathic concern are special cases of the kama muta emotion. This is consistent with the work of other scholars who have suggested that empathy or empathic concern is related to being moved (e.g., Baron-Cohen and Wheelwright, 2004; Menninghaus et al., 2015).

In contrast, across the 16 studies we did not find any other subscales of the IRI that consistently predicted ratings of being moved by our stimuli. (Fantasy showed an effect in one of the eight studies in which it was measured; cf. Eerola et al., 2016). We replicated earlier findings by Davis (1983b) who also observed no association between state perspective taking and states labeled as *moved*. In sum, kama muta seems to be related to empathic concern but not to other cognitive or affective concepts of *empathy*.

One might wonder how surprising this result is, given that the empathic concern subscale asks items such as “I am often quite touched by things that I see happen”. However, note that most other items focus on situations of need, and that we also found a relation of that subscale to reported physical sensations (crying, felt warmth, goosebumps), which the scale does not ask about. As indicated in Footnote 2, above, when we computed each model with the empathic concern subscale after removing the item reading: “I am often quite touched by things that I see happen” for the 16 studies, the overall effect size was not significantly different, *r* = 0.32 [0.26, 0.39]. It thus seems that both trait and state empathic concern are much broader phenomena than previously assumed.

## Empathic Concern as a Special Case of Kama Muta

We posit that sudden intensifications in communal sharing relationships result in kama muta, an emotional state typically labeled by English speakers as *being moved* or *touched* (Fiske et al., 2016; Schubert et al., 2016). There are two main reasons why we adopt kama muta as our theoretical construct instead of other conceptualizations of *being moved* experiences. First, previous conceptualizations have relied on the English vernacular label as a scientific construct (e.g., Cova and Deonna, 2014; Menninghaus et al., 2015). We think it is problematic to denote a construct with its linguistic label from one particular vernacular language, because many languages have different or no directly corresponding lexemes, and it is ethnocentric to assume that the English lexemes are the valid ones. Second, vernacular lexemes are used imprecisely, inconsistently, and differently in different dialects and historical periods. We believe that people experience kama muta in many contexts where they do not label the experiences ‘being moved,’ including in cultures whose languages have no specific or definite term for the emotion. Even in English, kama muta might sometimes be labeled differently than *being moved* or *being touched;* for example, as *nostalgia*, *feeling patriotic*, or religious *ecstasy*. Conversely, it is important to note that the specific vernacular terms such as *being moved* or *being touched* may sometimes also be used to refer to other experiences not captured by the kama muta model, *totum pro parte*. In sum, vernacular feeling terms make for imprecise psychological concepts because usage varies across individuals, situations, and cultures, and they may neither include all instances of the concept nor be exclusively used for them. Instead, feeling terms should be used as operationalizations of the feeling component of emotions. The kama muta model resolves these issues.

The state of empathic concern or sympathy as defined by Batson et al. (1987) is caused by observing other individuals in need. The question is how neediness constitutes a sudden intensification of communal sharing. We can think of two possible explanations. First, the common denominator seems to be the appeal to engage in a communal sharing relationship – when that appeal is perceived, it seems to be experienced as a suddenly intensifying relation, causing the emotion. Of course, communal sharing can also suddenly intensify when one’s toddler says “I love you, mom”; when one reunites with a loved one after a long separation; when one feels the love of a deity; when one feels patriotic pride for one’s nation (Fiske et al., 2017; Seibt et al., 2017). We assume that the appeal itself constitutes a similar intensification of communal sharing.

An alternative to this link between neediness and intensification of communal sharing is an evoked identification. Imagine walking past a homeless person. One might suddenly identify with the person, perhaps feeling that one might easily become homeless oneself, or simply feeling that suffering is a shared aspect of everyone’s humanity. If such an intensification of communality is strong enough it might result in feelings of kama muta (Schubert et al., 2016). This idea is consistent with other scholars’ observations about the close links between communal sharing and sympathy or empathic concern (Fiske, 1991; Pinker, 2011; Rai and Fiske, 2011; Iannone, 2014; Gaspar, 2016).

In sum, there are two possibilities: the appeal itself could constitute the sudden intensification or the evoked identification with the needy person could play a role. We have to leave it to future work to determine which of these two possibilities contributes more to kama muta evoked by displays of neediness.

The overlap of kama muta and empathic concern is further supported by the fact that both are often described in terms of experiences of “warmth” (Batson et al., 1987; Schubert et al., 2016). Physical warmth has been directly linked to communal sharing (IJzerman et al., 2015), and the participants in the present 16 studies on average reported increased feelings of warmth in their body when they also reported feeling *moved* or *touched* (Seibt et al., 2017, Seibt et al., unpublished). In addition, empathic concern also correlated positively with reported sensations of weeping and chills, though to a smaller degree. Chest or bodily warmth, moist eyes or tears, and goosebumps or chills have been identified as common physiological reactions to kama muta (Schubert et al., 2016).

Some authors have argued that empathic concern consists of two distinct states: *sympathy* and *tenderness* (Niezink et al., 2012). While sympathy is expected to occur in response to the acute needs of other individuals, tenderness is thought to be related to targets who are intrinsically vulnerable, especially cute ones. Two studies in the present investigation included cute stimuli that were not in any perceptive danger (Study 7 and 14) and effect sizes were not appreciably lower. Niezink et al. (2012) identified these two aspects in factor analyses of the state empathic concern scale, finding that the two correlated around, *r* = 0.48. The present findings cannot provide systematic evidence that only one or both of these concepts are related to kama muta. In any case, the kama muta concept includes both the aspect of *feeling moved* that is related to sympathy and the quality of warmth that corresponds to tenderness.

The present findings also suggested a relation between empathic concern and states of sadness. We believe that kama muta itself is a purely positive emotion, but can occur together with other emotions, such as sadness at loss or separation, for example. Firm conclusions are difficult if participants have to judge whole episodes that may involve several developing emotions over time. It is not possible to judge from the current studies whether the correlation between empathic concern and sadness is as strong as, or weaker than, the relation between empathic concern and kama muta. How can it be explained if we assume that empathic concern is part of the broader, positive kama muta emotion construct? As argued above, although kama muta has been reported to be generally positive, participants have characterized some experiences as having some negativity when summary judgments of whole episodes are elicited (Hanich et al., 2014; Seibt et al., unpublished). Continuously collected reports of feeling moved, sadness, and happiness suggest that sadness can, but does not always, co-occur with feeling moved: Schubert et al. (2016) showed participants one video clip including two orphans, one blind and one deaf, embarking on a journey to visit the grave of the mother of one of the children. One set of participants’ continuous ratings of sadness while watching the video cross-correlated highly with another set of participant’s ratings of being moved at the same points in time. However, for the five other clips utilized in the study, sadness and being moved showed distinct time curves that were not consistently correlated, while being moved and happiness cross-correlated positively in all five videos. In the studies included in the current meta-analysis, participants always gave summary judgments after the whole emotional episode concluded. We believe the most parsimonious explanation of the link between empathic concern and sadness is that the observed sadness reflects either the perceived background of loss against which kama muta is felt, or the realization that the increased communal sharing relation cannot be implemented (as it is the case in the videos with the two orphans). In addition, some videos showed protagonists who were sad in the middle of the episode, before ultimately being happily communal in the end. For those videos, participants’ reports of sadness may have reflected how they felt when the participants were sad, before the participants felt kama muta when a communal sharing relationship suddenly intensified at the end.

In general, empathic concern has been found to be positively valenced (Batson et al., 1987), similar to kama muta (Schubert et al., 2016; Seibt et al., unpublished). This observation might also explain the positive correlation of empathic concern and state happiness that was observed in one study. The finding that a general disposition of empathic concern or to feel compassion increases feelings of happiness has been observed in previous studies (e.g., Mongrain et al., 2011). The positive correlation of happiness and empathic concern may be based on the fact that happiness reflects the general positive valence of the kama muta state. This interpretation is in line with the finding that happiness indicates more a stable emotional background rather than discrete and short-lived emotional episodes that were the focus of the present studies (Gruber et al., 2013).

The present findings cannot quite exclude the possibility that empathic concern is associated with a general tendency of emotionality as discussed by Davis (1983a), in the sense that it increases the adoption of any emotion one sees in another person. While we found a number of emotions such as awe, fear or amusement that were not related to empathic concern, sadness and happiness were associated. It seems most likely to us that the scale measuring trait empathic concern does not predict the adoption of just any emotion seen in another, but is rather specific to social emotions linked to communal sharing. Future work should test this directly. More generally, it should be noted that the current work investigates the correlates of an established scale by linking it to a theorized emotion. Our results can inform interpretation of data previously collected with the scale, but to some extent they also suggest that the empathic concern scale could fruitfully be revised or reconstructed with reference to a more comprehensive and precise theory.

### Kama Muta and the Empathy-Altruism Link

Batson and colleagues have argued that state empathic concern causes increased motivation and intention to help altruistically (Coke et al., 1978; Batson et al., 1981, 2007; see also Jordan et al., 2016; Tusche et al., 2016). The authors have termed this the *empathy–altruism link* and have provided a considerable amount of empirical evidence to support it. Similarly, feelings labeled as *being moved* have been empirically shown to increase altruistic behavior (Freeman et al., 2009; Schnall et al., 2010; Thomson and Siegel, 2013). In one study by Schnall et al. (2010) participants were more likely to take part in an unpaid study after watching a moving video in contrast to a neutral video. Although the authors discuss their findings in light of the *elevation* framework, they measured, amongst other items such as *uplifted* or *warm chest*, feelings of *being moved*. Being moved and altruistic helping have also been associated on a theoretical basis (e.g., Baron-Cohen and Wheelwright, 2004; Menninghaus et al., 2015). For Baron-Cohen and Wheelwright the motivation to help is a part of sympathy, which they equate with feelings of being moved and touched.

The kama muta model argues that experiencing kama muta motivates individuals to affectively devote and morally commit to communal sharing relationships (Fiske et al., 2016). Communal sharing implies and requires helping those whom one includes in the ‘we’ of the relationship. The kama muta model can therefore theoretically account for the empathy–altruism link. However, only one study has systematically tested the motivations that grow out of kama muta so far (Zickfeld, 2015).

Our initial review of the literature on *empathy* shows that the same vernacular term is used to refer to a great many scientific constructs. We think that the term *empathy* is in many ways too broad and diffuse, and its use is too inconsistent. For greater precision and comparability, we propose that research should focus on clearly defined components such as perspective taking, emotional contagion, or, as in the present studies, empathic concern. The current studies have provided evidence that empathic concern predicts a class of phenomena much broader that it was originally intended to: kama muta responses not just to neediness, but to other affordance for sudden intensification of communal sharing. The resultant emotion is characterized not only by compassionate motivation to care for others, but also by warm feelings in the chest or elsewhere, moist eyes or tears, and goosebumps or chills. Future studies can and should strive to investigate *why* that is the case – what differentiates people high in empathic concern from others that makes them disposed to react emotionally in this way?

In sum, trait empathic concern, as measured by the subscale of the IRI (Davis, 1980), is a disposition to experience sympathy for those in need (Batson, 1990). Sympathy for the needy is a special case of communal sharing, a relation of loving kindness (Fiske, 2004). The sudden intensification of communal sharing that occurs when one feels loving kindness for a needy person is a special case of the general phenomenon of sudden intensification of communal sharing evoked by a wide variety of practices, narratives, and institutions. Based on the observed positive correlation we argue that empathic concern might be part of a more general communal emotion that is often evoked by mystical religious ecstasy, reunions or hearing Martin Luther King, Jr.’s “I have a dream” speech: kama muta.

## Ethics Statement

This study was carried out in accordance with the recommendations of the IRB of the University of Oslo with written informed consent from all subjects. All subjects gave written informed consent in accordance with the Declaration of Helsinki. The protocol was approved by the IRB of the University of Oslo.

## Author Contributions

All authors conceived, designed and conducted the studies, JZ conducted the statistical analyses, JZ and TS wrote the first draft, and all authors revised the final manuscript.

## Conflict of Interest Statement

The authors declare that the research was conducted in the absence of any commercial or financial relationships that could be construed as a potential conflict of interest.

## Footnotes

1 https://osf.io/nzsuj/

2 https://osf.io/9fr73/

3 Originally 3246 participants were sampled in total. Three hundred and twenty-eight participants were excluded; all exclusions were done according to *a priori* criteria and before importing data. Exclusion criteria for the respective studies are detailed in the Supplementary Material.

4 In addition, we computed each model with the empathic concern subscale without the item reading: “I am often quite touched by things that I see happen” for the 16 studies. The overall effect size was not significantly different, *r* = 0.32 [0.26, 0.39].

5 Note that our meta-analysis included only four published studies. Because the majority consisted of unpublished studies we did not apply methods correcting for publication bias.

6 We also tested for measurement invariance across the different studies and countries. Results generally suggested support for metric invariance and can be found in the Supplementary Materials.

7 Note that these analyses were not part of the pre-registration of Study 16, but added later for exploratory reasons.
