# Supplementary material for: Empathic Concern Is Part of a More General Communal Emotion
Source: Front Psychol. 2017 May 10;8:723. doi: 10.3389/fpsyg.2017.00723 (PMC5423947; doi:10.3389/fpsyg.2017.00723)
Supplement: Supplementary file 1 [file Data_Sheet_1.docx]

Supplementary Material

Empathic Concern is Part of A More General Communal Emotion

Janis H. Zickfeld*, Thomas W. Schubert, Beate Seibt, Alan P. Fiske

*Correspondence: Janis H. Zickfeld: j.h.zickfeld@psykologi.uio.no

**Method**

In addition to the presented measures in the main text, the sixteen studies assessed a number of different variables and used various stimuli in order to induce various affective states.

**Study 1.** In the beginning, participants were presented with the short

form UCLA loneliness scale (USL-8, Hays and DiMatteo, 1987). The scale consists of

eight items targeting general dispositions of loneliness (e.g. ‘’I lack companion

ship’’; ‘’I feel isolation from others’’). Questions were completed on a 4-point

scale including labels *never, rarely, sometimes* and *often*. Afterwards participants were randomly presented with either 5 or 10 out of 21 narratives. Narratives were written by a different sample of participants in a pilot study asking for studies that would *move* or *touch* other individuals. After each narrative participants completed the same items.

First, the emotions elicited by the narrative were assessed on a 7-point scale ranging from *not at all* to *very much*. In addition to items on moved and touched, participants were asked to indicate feelings of awe, happiness, sadness, gratitude, admiration and helplessness. Then participants were asked to complete five items assessing possible appraisals as adapted from (Seibt et al., 2017). The items included statements about moral right actions, characters coming closer together, inclusion of a person, overcoming of obstacles and a person becoming more human. These items were rated on a 5-point scale ranging from *not at all* to *to a high degree*. Afterwards, participants were asked to indicate their physiological reactions on a 5-point scale anchored at *not at all* and *very much*. Physiological symptoms included: tears/moist eyes, warm chest, chills/goosebumps, choking feeling of constriction in throat, I smiled, I sighed or made a sound like ‘Ahh’, I put my hand to my chest or face, and other.

Finally, participants completed the mini-IPIP scales (Donnellan et al., 2006) containing a 20 item scale assessing the Big Five factors of personality. Then participants completed the IRI scale (Davis, 1980) and demographics.

In total, 81 participants were recruited. One participant was excluded due to not completing the questionnaire.

**Study 2.** In the beginning, participants were presented with four items included to assess mood effects. The items asked ‘’How is your mood at the moment?’’ (bad mood – good mood), ‘’Do you feel tired or energetic?’’ (tired – energetic), ‘’Do you feel irritated?’’ (not at all irritated – very much irritated), and ‘’Do you feel sad or cheerful?’’ (sad – cheerful). All items were assessed on 5-point scales.

Then participants were always shown the same *anchor* video (*Thai medicine*) adapted from (Schubert et al., 2016). Secondly, participants were either presented with an original or manipulated version of two different videos (*Christian the Lion* and *Orphans*). If the original Christian the Lion video was presented first, the manipulated Orphans video was presented afterwards and vice versa, resulting in four different combinations. After each of the three videos participants completed the same items. The manipulated version of both videos tried to exclude instances of communal sharing conformations such as touch, eye contact, commensalism (e.g., Fiske, 2004) while still keeping the narrative of the video. The edited Christian the Lion video showed the protagonists next to each other, but not hugging and caressing as in the original version. The edited Orphans video excluded the main obstacles introduced in the original video.

After completing two items on how *moved* or *touched* the video made feel, participants were asked to judge whether the characters in the video *grew strongly apart* or *got strongly closer* (7-point scale). Participants were also asked whether the characters in the video expressed love or affection towards each other and whether they helped or shared on a 5-point scale ranging from *not at all* to *clearly*. Then participants completed the same appraisal and physiology items as in Study 1. The physiological items were rated on a 3-point scale from *not at all* to *clearly*. In the end participants were asked whether they watched the sound, had seen the video before or had problems with video playback.

Finally, we presented the IRI scale, the BFI-10 (Rammstedt and John, 2007) and questions assessing demographic information. In total 156 participants were recruited and four were excluded due to missing data (>50%).

**Study 3.** In the third study, participants were shown three different videos (*Thai medicine, Math* and *Dad*) in random order. After each video participants completed an unpublished early version of the unpublished Kama Muta Multiplex Scale (KAMMUS; see Appendix B). The KAMMUS consists of four different subscales respectively assessing physiology, appraisals, motivations and vernacular emotion ratings. All ratings are completed on 7-point scales ranging from *not at all* to *a lot*. After each video, participants were also asked whether they had seen the video before, listened to the audio and whether they had technical problems.

In the end participants were presented with the IRI scale and the mini IPIP, as well as items targeting demographic information.

In total 95 participants were sampled. Two participants were excluded based on specifying technical difficulties or a video timer revealing that they did not watch one or more videos.

**Study 4.** Participants were presented with one out of six different videos (*Marriage, I’m just a human being, Thai altruism, Dad surprise, Disabled surprise* and *Sister*). Then participants were asked to complete the KAMMUS scale as in Study 3. Before completing questions about video playback, sound and whether they had seen it before participants were asked whether the video reminded them of similar own relationships or situations and whether the social relations or situations depicted had been important for them as well. These four items were completed on a 7-point scale ranging from *not at all* to *very much*.

Finally, participants were presented with the IRI scale (only PT and EC), the Short Dark Triad (SD3, Jones and Paulhus, 2014), measuring Machiavellianism, narcissism and psychopathy and items assessing demographics. In total 140 participants were recruited while none were excluded.

**Study 5.** In the beginning participants completed the same mood items as in Study 2. They were then presented with an *anchor* video (*Dog homecoming*) and completed items on how moved and touched they were and whether the relations between the characters got stronger or not. These items were completed on 5-point scales. Then participants completed the same physiological symptoms list as in Study 1 using the same scale as in Study 2.

Then participants were randomly allocated to one of three different groups. All read a short description about a young girl and her father who was stationed in Iraq. The independent variable manipulated the last time the two had seen each other and whether their last meeting ended in a fight (*short vs. long vs. long problematic*). After answering some manipulation check questions and how close the participants thought the two characters were before the reunion the participants watched a video clip ostensibly presenting the same characters. They were then asked how close they thought the two were after the reunion (5-point scale) and items on how moved and touched the video made them. Participants then completed the same physiological items and scales as in Study 2 and questions about sound, playback problems and familiarity.

Finally, participants were asked to complete the IRI, BFI-10, and demographic items.

In total 138 participants were recruited. Twenty-three participants were eventually filtered out either due to completing no demographics, failing to correctly answer a question targeted at the manipulation or because they indicated having seen the video before.

**Study 6.** In the beginning the participants completed the USL-8 and the same mood items as in the previous studies. They were then presented with a picture of a man and randomly assigned to one of two conditions. In one condition participants were told that the person was just released from prison after a bank robbery, while others were told that he just got released from prison. Participants then completed the IOS scale (Aron et al., 1992) with regard to how much they identified with him. They also completed items on how much compassion they felt, how disgusted they were by him and whether they thought the target person was a person that would normally act morally or ethically right. All items were presented on 7-point scales ranging from *not at all* to *extremely*.

Participants then watched a clip depicting the man reuniting with his dog (*Dog homecoming*). They were asked how moved and touched the video made them and also asked to fill out a pictorial measure showing on the one side a neutral face and on the other a crying person that puts a hand to the chest. Then they completed items on how positive and negative the video was, as well as whether they were sad, happy, disgusted by the person or do identify with him (all 7-point scales and identification with the IOS measure).

Afterwards, participants indicated whether characters in the video expressed love or affection on a 5-point scale (*not at all* – *clearly*) and completed the five appraisal items and the physiological symptoms list and scale as used in Study 1. After reporting items on sound, playback problems and familiarity participants were asked to complete the IRI (PT and EC only) and demographic information.

In total 168 participants were recruited. Finally, 57 participants were excluded based on indicating that they did not want their data to be analyzed (educational purposes only) or because of failing to complete the questionnaire.

**Study 7.** Participants were randomly presented with either a cute or non-cute (neutral) video first. After watching a cute video first the participants watched a non-cute video afterwards and vice versa. For each video type one video was randomly chosen from a pool of four different videos (non-cute: *octopus, monkey, fish, shark;* cute: *rabbit, kitten ball, kitten stick, kitten bowl*). After each video the participants completed a scale targeting cuteness. The scale included 12 items such as ‘’it is adorable’’ or ‘’it is cuddly’’ on a 7-point scale anchored at *not at all* and *a lot*. Then participants completed the KAMMUS scale as in previous studies with an adjusted version of the fourth subscale only including items on heartwarming, moved, touched, sad, scared and disgusted.

Finally, participants completed the empathic concern subscale of the IRI and items targeting demographic information.

In total 229 participants were recruited, with 10 being eventually excluded because of not watching a video or due to failing age requirements.

**Study 8.** Participants were asked to only take part in the study if they experienced *happy tears* on the previous day (the study was conducted one day after Valentines day). They were then asked to remember the event in which they had happy tears and describe it in three or four sentences. Afterwards participants completed the KAMMUS with regard to the event. Finally, the IRI and items assessing demographics were presented. In total 54 participants were recruited; none was excluded.

**Study 9.** Study 9 was in general a duplicate of Study 8. Instead of happy tears participants were asked to remember and describe an event in which ‘’a relationship grew stronger’’. After describing the event in three to four sentences the same measures as in Study 8 were presented.

In total 53 participants were recruited. One participant was excluded due to failing to provide a description of an event.

**Study 10.** In the beginning participants were asked to provide both the name of a close friend and a distant acquaintance. Afterwards participants were randomly assigned to one out of three audio narratives. Each narrative told the story of Laura who owns a small coffee shop. In the moving condition, Laura altruistically helped homeless people by giving them food; in the amusement condition Laura told about a rude customer falling into a pie; in the neutral condition Laura described the interior of the café.

After listening to the story participants were asked to rate their feelings with items assessing moved, touched, amused, happy, sad and uplifted. All items were answered on a 7-point scale anchored at *not at all* and *very much*. Participants then completed the same physiological symptoms list as in Study 1 with moist eyes being an own item now. After indicating the familiarity of the story participants were asked to estimate their room temperature. This item was embedded among other unrelated measures as used in previous research (IJzerman and Semin, 2010). Participants were then asked to complete the Communal Strength scale (Mills et al., 2004) with regard to the main protagonist of the story (Laura). The scale includes items such as ‘’how far would you be willing to vist xy?’’ or ‘’how reluctant would you be to sacrifice for xy?’’ rated on a 10-point scale (*nothing at all* – *extremely large*).

Participants then completed the IOS measure with regard to the close or distant target specified in the beginning (this was randomly assigned between participants). Then participants completed the IRI scale (without FS) and the revised Adult Attachment Scale (AAS, Collins, 1996), as well as demographic information.

In total 424 participants were recruited. Finally 24 individuals were excluded because they provided residing in a country different than the US.

**Study 11.** Similarly to Study 10, participants were asked to indicate the first name of both a close friend and a distant acquaintance in the beginning. Participants were then randomly allocated to a moving, amusing or neutral condition. In each condition participants were asked to remember either a moving/touching or amusing moment with their mother and write it down with at least 200 characters. In the neutral condition, participants were asked to describe a normal work day of their mother.

Then emotion items and physiological symptoms were rated using the same scales as in Study 10. Afterwards the Communal Strength scale and the IOS measure were completed for both the mother and the father of the participant. Participants also rated how much they would be willing to hug, call or share money with both targets. Then participants completed the IOS measure with regard to the close friend or distant acquaintance. Again, this was randomly allocated between participants.

Finally, participants completed the IRI (without FS), the AAS and demographic information.

In total 323 participants were recruited. Eighteen participants were eventually excluded due to indicating another country of residency than the US. One case was excluded due to missing answers for all items.

**Study 12.** In the beginning, participants completed the USL-8 and were then randomly presented with four different videos targeted at eliciting fear (from *The Shining*), happiness (*Singing in the rain*), sadness (*Two Orphans Cut*), and being moved (*Thai Medicine*). After each video participants completed a number of affective items: moved, touched, sad, happy and anxious on a 7-point scale (*not at all* – *extremely*). They were then asked to what degree they identified with the character(s) in the video rated on the same type of scale. In addition, we also asked whether characters in the video expressed love or affection as assessed on the same scale as in earlier studies (e.g. Study 2).

Participants were then presented with the appraisal and physiological symptoms list as in Study 1, both employing a 5-point scale. Then participants were asked to indicate problems with video playback, sound and familiarity.

In the end, participants were asked to complete the IRI scale, presented with a positive stimulus because of the fear and sad inducing videos and finally items targeting demographic information.

In total 252 participants were recruited. Eventually 32 individuals were excluded based on a timer suggesting they did not watch the whole video or because they indicated that they did not want their data to be analyzed (educational purposes only).

**Study 13.** In the thirteenth study participants were asked to remember an episode or situation in which they got moist eyes or even shed tears because of either a positive or a negative feeling. The presentation of the positive and negative instructions was randomized across participants. After writing down each of the descriptions participants completed a list of affective states targeting their emotional reaction during the remembered episode. The list included: moved, touched, happy, sad, angry, anxious, admiration, uplifted, awe, pride and unity. The items were rated on a 7-point scale anchored at *not at all* and *extremely*.

Then participants rated the appraisal list as employed in Study 1. This time however on a 7-point scale ranging from *on the contrary = -3* to *to a high degree = 3*. Afterwards the same physiological symptoms were presented as in Study 10 employing the same scale. In the end participants completed the IRI scale, the AAS and finally demographics.

In total 241 participants were sampled. Four individuals were excluded because they failed to provide a narrative for either the positive or negative tears.

**Study 14.** In Study 14 participants were randomly presented with one out of eight different videos and afterwards completed a number of measures. After the video participants first filled out the KAMMUS scale as in some of the previous studies. Then participants completed two measures of whether they felt member of the same group as the protagonist of the video on a 7-point scale (*not at all*, *very much*; (Gaertner et al., 1989). Afterwards followed an unpublished scale assessing humanization presenting three items assessing the perception of humanness of the protagonist completed on a 7-point scale. Then participants completed the common in-group measure by (Vezzali et al., 2015). After that, participants where asked whether the protagonist of the video was *mostly an individual* (1) or on the same 7-point scale *mostly a member of a group* (7). Then they completed an empathy measure by (Capozza et al., 2013) on a 7-point scale (*not at all, a lot*). Afterwards participants were presented with the communal sharing measure by (Haslam, 1994) including six statements on a 7-point scale. Then participants rated their own and the protagonist’s overlap with human kind using a scale by (Schubert and Otten, 2002). Afterwards participants completed the feeling thermometer for their in-group (US Americans; Haddock et al., 1993). Then they engaged in rating a number of traits on whether they were seen as part of human nature or uniquely human on 7-point scales. The traits were adapted from (Miranda et al., 2014). After that participants completed the same feeling thermometer for the protagonist’s group (Haddock et al., 1993). Then participants were presented with a measure assessing blatant humanization on a 7-point scale (Kteily and Bruneau, 2017). Finally, participants completed the empathic concern subscale of the IRI. In total 386 participants were recruited and 73 participants were excluded based on not watching the whole video (which was controlled with a timer) or providing a majority of missing answers (>50%).

**Study 15.** In Study 15 participants were presented with two different videos. Videos differed on what type of animal they showed (cat vs. dog) and whether these animals were alone or accompanied by another animal of their species. Participants were randomly presented with a crossed selection of those videos. That is a participants first watching the *cat alone* video afterwards received the *dog companion* video and vice versa resulting in four different combinations. After each video participants completed the same measures.

First, participants completed a similar cuteness scale as in Study 7. The scale included ten items (e.g., “The video was adorable”) that were rated on a 7-point scale (*not at all*, *a lot*). Then participants were presented with the KAMMUS as in previous studies. After both videos participants completed the empathic concern subscale of the IRI and demographic questions. In total 201 participants completed the study (402) cases and 63 were excluded based on failing to watch the videos.

**Study 16.** After being provided with informed consent and accepting the terms by continuing with the survey participants were presented with the IRI including all four subscales. Afterwards participants were randomly allocated to one out of four emotion induction conditions: *being moved, awe, amusement* and *sadness*. For each condition a set of three different video stimuli was available, from which one was chosen randomly. Some of the video stimuli were based on earlier research (Schaefer, Nils, Sanchez, & Philippot, 2010; Schubert et al., 2016; Seibt et al., 2016). All participants completed all four conditions as well as the same measures afterwards.

After each of the four videos, participants were presented with the KAMMUS (Kama Muta Multiplex Scale) assessing different parts of the kama muta framework. The first part consisted of physiology and action items including: *moist eyes, tears, goosebumps or hair standing up, chills or shivers, a warm feeling in the center of the chest, some feeling in the center of the chest, choked up, a lump in the throat, I had difficulty speaking, I put one or both hands to my chest, I took a deep breath or held my breath, I sad something like ‘awww’, (I felt) buoyant or light, (I felt) refreshed, energized or exhilarated*. In addition, the first part included five filler items: *sneezed or felt like sneezing headache, laughed or giggled, sick to my stomach* and *dizzy*.

The second part consisted of 12 different appraisal items such as *I observed, heard, or read about an incredible bond* or *I observed, heard, or read about a phenomenal feeling when someone is being appreciated*. The third part consisted of 7 motivational items such as *I wanted to hug someone* or *I felt more strongly committed to a relationship*. Both sections were complemented with filler items.

The final part assessed emotion labels. Items on *heartwarming, moved, touched, nostalgic moment, poignant experience, felt a part of something larger than myself, felt in love, sad, great respect* and *proud* were presented. We added items on *awe,* and *amusing*.

All sections were rated on a 7-point scale anchored at *0 = not at all* and *6 = a lot*.

Participants were also asked whether they had problem with video playback, whether they had seen the movie before and whether they listened to the sound. For exploratory reasons we also included the Kama Muta Frequency Scale (KAMF) a scale containing 7 items intended to measure the general proneness to feeling moved or touched. In addition we also included the Southampton Nostalgia Scale (SNS; Sedikides et al., 2008). Finally, the participants completed demographic information including gender, age, nationality, relationship status, number of children and whether they have pets.

In total 289 participants were recruited. Eleven cases were excluded based on a timer suggesting that participants did not watch the whole video.

**Results**

**Measurement Invariance.** The importance of testing for measurement invariance, whether the same items actually measure the same latent construct across groups (e.g., sex, nationality), has been emphasized for cross-cultural comparisons (Van De Schoot et al., 2015; van de Vijver and Tanzer, 2004). It has also been argued that measurement invariance should be tested before meta-analyzing different studies (Fabrigar and Wegener, 2016). We therefore explored whether the seven empathic concern items measured the same latent constructs across (a) all 16 studies and across (b) both countries.

The literature has typically summarized three different levels of measurement invariance: *configural, metric* and *scalar* invariance (Davidov et al., 2014; Van de Schoot et al., 2012). Configural invariance refers to the lowest level of achieving the same factor structure across groups, metric to the observation of similar factor loadings across groups and finally scalar to similar intercepts across groups. Which type of invariance is required is debated. A number of researchers have argued that achieving scalar invariance is impractical, especially in studies containing a large number of participants and groups (Byrne et al., 1989). We therefore opted for a less conservative strategy testing measurement invariance using the *alignment* method (Asparouhov and Muthén, 2014; Marsh et al., 2017). Metric invariance has also been argued to be sufficient in order to compare correlation coefficients across groups, while most scholars argue that scalar invariance is needed to compare means across groups meaningfully (Steinmetz et al., 2008). Because of our meta-analytically approach we therefore decided that metric invariance should suffice our needs.

The alignment procedure was implemented using Mplus (Muthén and Muthén, 2010). We first tested for measurement invariance across all 16 studies. We used all seven items of the empathic concern subscale including three reversed scored items. Configural invariance held for all studies except for Study 14. The test procedure reported differences in factor loadings only in Study 14 (item #1) and Study 1 (#3 and #7) failing for approximate invariance (Supplementary Table 2). Hence, we repeated the random effects meta-analysis model excluding these two studies. The result was not considerably different from our original effect size, *r* = .34 [.27, .40]. In addition, we also tested scalar invariance. The approximation for invariance of intercepts failed for Study 2 (#6 and #7), Study 6 (#4 and #7), Study 7 (#1 and #2), Study 10 (#6), and Study 15 (#4 and #7). Repeating the random effects model excluding those studies did not yield a significantly different estimate, *r* = .34 [.26, .41].

Finally, we repeated the procedure assessing measurement invariance across the two countries (US vs. NO). First of all, configural invariance was established. Across both groups all items loaded on the same factor. In addition, we also observed metric invariance. Factor loadings across the two groups did not vary significantly from each other. Finally, we could not establish scalar invariance. The intercepts for item #4, #5, and #7 differed significantly across both countries (Supplementary Table 3).

**Pre-registered Analyses Study 16**

For Study 16 we pre-registered a number of analyses that are presented in more depth below.

**Differences among videos and participants.** First of all, we explored whether participants perceived the different conditions as they were intended. All of the following analyses were conducted using hierarchical mixed models. We conducted a model using the MIXED command in SPSS 24 with the emotion rating as DV, a factor coding for type of emotion rating (moved vs. awe vs. amusement vs. sadness), a factor coding for type of video type (moved vs. awe vs. amusement vs. sadness) and a factor coding for gender. Intercepts were allowed to vary randomly across participants and video. We observed a number of main effects and interactions. First, feeling moved ratings were strongest, *F*(3, 2837) = 4.81, *p* = .002, as well as (any type of) ratings for the moving video, *F*(3, 3057) = 171.78, *p* < .001. Gender indicated an interaction with type of video, *F*(3, 3057) = 8.16, *p* < .001, and type of emotion ratings, *F*(3, 2837) = 19.40, *p* < .001. Emotion ratings of awe and amusement were higher for males, while ratings of sadness and feeling moved were higher for females. The type of video interaction with gender showed the same pattern. Emotion ratings did differ for the different types of videos, *F*(9, 2837) = 162.55, *p* < .001. The amusement rating was highest for the amusing video, the sadness rating for the sad video, the awe rating for the awe video and the feeling moved rating for the moving video (see Table 4). Finally, we also observed a weak three-way interaction with measurement type, video type and gender, *F*(9, 2837) = 1.96, *p* = .040. Gender did not indicate a significant main effect.

**Directly comparing empathic concern with the four affective states.** In another analysis we compared the four affective states for their association with empathic concern. Emotion rating was used as DV, a factor coding for type of emotion rating and type of emotion condition (video), as well as the score on empathic concern were employed as predictors. We observed the expected type of emotion rating x empathic concern interaction, *F*(3, 2847) = 7.11, *p* < .001. Ratings of feeling moved were higher for high values of empathic concern, while lower for low values of empathic concern (Figure 1). This was not the case for amusement and awe ratings, but for sadness ratings. The effect for feeling moved ratings was highest though. Finally, we also observed a three-way interaction of type of emotion rating x type of video x empathic concern, *F*(9, 2847) = 3.36, *p* < .001.

Tables

Supplementary Table 1. Overview of all studies and the number of participants that were used for the final random effects models for the measures of feeling moved, tears, warmth and chills. Differences are mostly due to missing data.

| Study # | *n* (cases) | | | |
| --- | --- | --- | --- | --- |
|  | Feeling Moved | Tears | Warmth | Chills |
| 1 | 80 (605) | 80 (604) | 80 (602) | 80 (602) |
| 2 | 152 (456) | 152 (456) | 152 (456) | 152 (454) |
| 3 | 91 (243) | 91 (241) | 91 (243) | 91 (243) |
| 4 | 140 | 137 | 139 | 139 |
| 5 | 115 | 115 | 115 | 114 |
| 6 | 111 | 110 | 109 | 110 |
| 7 | 218 | 219 | 219 | 219 |
| 8 | 54 | 54 | 54 | 54 |
| 9 | 52 | 52 | 52 | 52 |
| 10 | 137 | 137 | 136 | 137 |
| 11 | 89 | 89 | 88 | 89 |
| 12 | 204 | 204 | 204 | 203 |
| 13 | 236 | 236 | 235 | 233 |
| 14 | 313 | 313 | 313 | 313 |
| 15 | 138 | 138 | 137 | 138 |
| 16 | 278 | 278 | 278 | 278 |
| Davis (1983b) | 158 | - | - | - |
| Eerola et al. (2016) | 102 | - | - | - |

Supplementary Table 2. Overview of invariance tests across all 16 studies for the empathic concern subscale. Numbers denote study number; parentheses indicate that the study failed invariance approximation for that item.

| Item # | Factor Loadings (Metric) | Intercepts (Scalar) |
| --- | --- | --- |
| 1 | 1 2 3 4 5 6 7 8 9 10 11 12 13 (14) 15 16 | 1 2 3 4 5 6 (7) 8 9 10 11 12 13 14 15 16 |
| 2 (r) | 1 2 3 4 5 6 7 8 9 10 11 12 13 14 15 16 | 1 2 3 4 5 6 (7) 8 9 10 11 12 13 14 15 16 |
| 3 | (1) 2 3 4 5 6 7 8 9 10 11 12 13 14 15 16 | 1 2 3 4 5 6 7 8 9 10 11 12 13 14 15 16 |
| 4 (r) | 1 2 3 4 5 6 7 8 9 10 11 12 13 14 15 16 | 1 2 3 4 5 (6) 7 8 9 10 11 12 13 14 (15) 16 |
| 5 (r) | 1 2 3 4 5 6 7 8 9 10 11 12 13 14 15 16 | 1 2 3 4 5 6 7 8 9 10 11 12 13 14 15 16 |
| 6 | 1 2 3 4 5 6 7 8 9 10 11 12 13 14 15 16 | 1 (2) 3 4 5 6 7 8 9 (10) 11 12 13 14 15 16 |
| 7 | (1) 2 3 4 5 6 7 8 9 10 11 12 13 14 15 16 | 1 (2) 3 4 5 (6) 7 8 9 10 11 12 13 14 (15) 16 |

*Note. (r)* denotes reverse coded items.

Supplementary Table 3. Overview of invariance tests across both countries for the empathic concern subscale. Numbers denote country (US: 0, NO: 1); parentheses indicate that the study failed invariance approximation for that item.

| Item # | Factor Loadings | Intercepts |
| --- | --- | --- |
| 1 | 0 1 | 0 1 |
| 2 (r) | 0 1 | 0 1 |
| 3 | 0 1 | 0 1 |
| 4 (r) | 0 1 | (0) (1) |
| 5 (r) | 0 1 | (0) (1) |
| 6 | 0 1 | 0 1 |
| 7 | 0 1 | (0) (1) |

*Note. (r)* denotes reverse coded items.

Supplementary Table 4. Overview of descriptives for the different videos and ratings in the pre-registered Study 16.

| Rating | Video (*M, SD*) | | | |
| --- | --- | --- | --- | --- |
|  | Amusement (*n* = 188) | Sadness (*n* = 196) | Awe (*n* = 131) | Moved (*n* = 278) |
| Amused | 3.81 (2.22) | 1.28 (.99) | 2.49 (1.87) | 2.97 (1.92) |
| Sad | 1.44 (1.01) | 4.96 (1.91) | 1.25 (.76) | 3.26 (2.04) |
| Awed | 1.56 (1.33) | 1.85 (1.50) | 3.82 (2.42) | 3.28 (2.15) |
| Feeling Moved | 1.38 (.90) | 2.66 (1.74) | 2.39 (1.50) | 5.31 (1.64) |

Figures


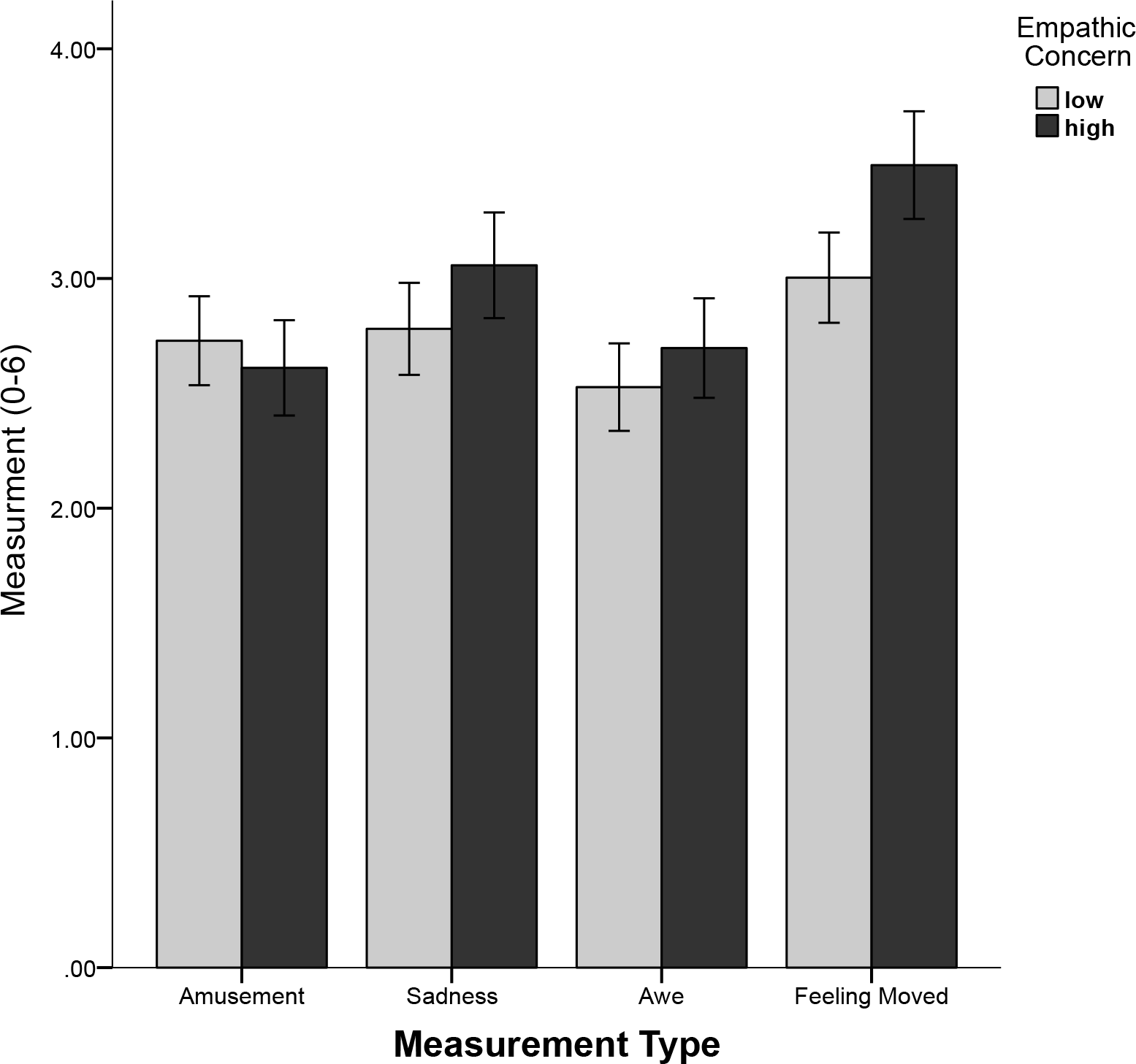


*Supplementary Figure 1.* Depiction of measurement type for low or high values of empathic concern (median split). Error bars represent 95% confidence intervals.

References

Aron, A., Aron, E. N., and Smollan, D. (1992). Inclusion of Other in the Self Scale and the structure of interpersonal closeness. *J. Pers. Soc. Psychol.* 63, 596–612. doi:10.1037/0022-3514.63.4.596.

Asparouhov, T., and Muthén, B. (2014). Multiple-group factor analysis alignment. *Struct. Equ. Model. Multidiscip. J.* 21, 495–508.

Byrne, B. M., Shavelson, R. J., and Muthén, B. (1989). Testing for the equivalence of factor covariance and mean structures: The issue of partial measurement invariance. *Psychol. Bull.* 105, 456.

Capozza, D., Falvo, R., Favara, I., and Trifiletti, E. (2013). The relationship between direct and indirect cross-group friendships and outgroup humanization: Emotional and cognitive mediators. *Test. Psychom. Methodol. Appl. Psychol.* 20, 383–398.

Collins, N. L. (1996). Working models of attachment: Implications for explanation, emotion, and behavior. *J. Pers. Soc. Psychol.* 71, 810–832. doi:10.1037/0022-3514.71.4.810.

Davidov, E., Meuleman, B., Cieciuch, J., Schmidt, P., and Billiet, J. (2014). Measurement equivalence in cross-national research. *Sociology* 40. Available at: http://www.annualreviews.org/eprint/dDDYVNPaTzsgSDbcT3y6/full/10.1146/annurev-soc-071913-043137 [Accessed September 11, 2016].

Davis, M. H. (1980). A multidimensional approach to individual differences in empathy. *JSAS Cat. Sel. Doc. Psychol.* 10, 85.

Donnellan, M. B., Oswald, F. L., Baird, B. M., and Lucas, R. E. (2006). The mini-IPIP scales: tiny-yet-effective measures of the Big Five factors of personality. *Psychol. Assess.* 18, 192.

Fabrigar, L. R., and Wegener, D. T. (2016). Conceptualizing and evaluating the replication of research results. *J. Exp. Soc. Psychol.* 66, 68–80. doi:10.1016/j.jesp.2015.07.009.

Fiske, A. P. (2004). “Relational models theory 2.0,” in *Relational Models Theory: A Contemporary Overview*, ed. N. Haslam (Mahwah, NJ: Erlbaum), 3–25.

Gaertner, S. L., Mann, J., Murrell, A., and Dovidio, J. F. (1989). Reducing intergroup bias: The benefits of recategorization. *J. Pers. Soc. Psychol.* 57, 239.

Haddock, G., Zanna, M. P., and Esses, V. M. (1993). Assessing the structure of prejudicial attitudes: The case of attitudes toward homosexuals. *J. Pers. Soc. Psychol.* 65, 1105.

Haslam, N. (1994). Categories of social relationship. *Cognition* 53, 59–90.

Hays, R. D., and DiMatteo, M. R. (1987). A short-form measure of loneliness. *J. Pers. Assess.* 51, 69–81.

IJzerman, H., and Semin, G. R. (2010). Temperature perceptions as a ground for social proximity. *J. Exp. Soc. Psychol.* 46, 867–873.

Jones, D. N., and Paulhus, D. L. (2014). Introducing the short dark triad (SD3) a brief measure of dark personality traits. *Assessment* 21, 28–41.

Kteily, N., and Bruneau, E. (2017). Backlash: The Politics and Real-World Consequences of Minority Group Dehumanization. *Pers. Soc. Psychol. Bull.* 43, 87–104.

Marsh, H. W., Guo, J., Parker, P. D., Nagengast, B., Asparouhov, T., Muthén, B., et al. (2017). What to do When Scalar Invariance Fails: The Extended Alignment Method for Multi-Group Factor Analysis Comparison of Latent Means Across Many Groups. Available at: http://psycnet.apa.org/psycinfo/2017-01642-001/ [Accessed February 10, 2017].

Mills, J., Clark, M. S., Ford, T. E., and Johnson, M. (2004). Measurement of communal strength. *Pers. Relatsh.* 11, 213–230.

Miranda, M., Gouveia-Pereira, M., and Vaes, J. (2014). When in Rome… Identification and acculturation strategies among minority members moderate the dehumanisation of the majority outgroup. *Eur. J. Soc. Psychol.* 44, 327–336.

Muthén, L. K., and Muthén, B. O. (2010). *Mplus User’s Guide: Statistical Analysis with Latent Variables: Users Guide*. Los Angeles, CA: Muthén & Muthén.

Rammstedt, B., and John, O. P. (2007). Measuring personality in one minute or less: A 10-item short version of the Big Five Inventory in English and German. *J. Res. Personal.* 41, 203–212.

Schaefer, A., Nils, F., Sanchez, X., and Philippot, P. (2010). Assessing the effectiveness of a large database of emotion-eliciting films: A new tool for emotion researchers. *Cogn. Emot.* 24, 1153–1172. doi:10.1080/02699930903274322.

Schubert, T. W., and Otten, S. (2002). Overlap of self, ingroup, and outgroup: Pictorial measures of self-categorization. *Self Identity* 1, 353–376.

Schubert, T. W., Zickfeld, J. H., Seibt, B., and Fiske, A. P. (2016). Moment-to-Moment Changes in Being Moved Match Changes in Perceived Closeness, Weeping, Goosebumps, and Warmth: Time Series Analyses. *Cogn. Emot.* doi:https://doi.org/10.1080/02699931.2016.1268998.

Sedikides, C., Wildschut, T., Arndt, J., and Routledge, C. (2008). Nostalgia: Past, Present, and Future. *Curr. Dir. Psychol. Sci.* 17, 304–307. doi:10.1111/j.1467-8721.2008.00595.x.

Seibt, B., Schubert, T. W., Zickfeld, J. H., and Fiske, A. P. (2017). Interpersonal Closeness and Morality Predict Feelings of Being Moved. *Emotion*. doi:doi:10.1037/emo0000271.

Steinmetz, H., Schmidt, P., Tina-Booh, A., Wieczorek, S., and Schwartz, S. H. (2008). Testing measurement invariance using multigroup CFA: differences between educational groups in human values measurement. *Qual. Quant.* 43, 599. doi:10.1007/s11135-007-9143-x.

Van de Schoot, R., Lugtig, P., and Hox, J. (2012). A checklist for testing measurement invariance. *Eur. J. Dev. Psychol.* 9, 486–492.

Van De Schoot, R., Schmidt, P., De Beuckelaer, A., Lek, K., and Zondervan-Zwijnenburg, M. (2015). Editorial: Measurement Invariance. *Front. Psychol.* 6. Available at: http://www.ncbi.nlm.nih.gov/pmc/articles/PMC4516821/ [Accessed September 11, 2016].

van de Vijver, F., and Tanzer, N. K. (2004). Bias and equivalence in cross-cultural assessment: an overview. *Rev. Eur. Psychol. AppliquéeEuropean Rev. Appl. Psychol.* 54, 119–135. doi:10.1016/j.erap.2003.12.004.

Vezzali, L., Stathi, S., Crisp, R. J., Giovannini, D., Capozza, D., and Gaertner, S. L. (2015). Imagined intergroup contact and common ingroup identity. *Soc. Psychol.* Available at: http://econtent.hogrefe.com/doi/full/10.1027/1864-9335/a000242 [Accessed February 14, 2017].

Appendix

Appendix A.

**IRI (Interpersonal Reactivity Index, Davis, 1980)**

DOES NOT DESCRIBE ME WELL (1)

DESCRIBES ME VERY WELL (5)

1. I daydream and fantasize, with some regularity, about things that might happen to me. (FS)

2. I often have tender, concerned feelings for people less fortunate than me. (EC)

3. I sometimes find it difficult to see things from the “other guy’s” point of view. (PT) (-)

4. Sometimes I don’t feel very sorry for other people when they are having problems. (EC)

5. I really get involved with the feelings of the characters in a novel. (FS)

6. In emergency situations, I feel apprehensive and ill-at-ease. (PD)

7. I am usually objective when I watch a movie or play, and I don’t often get completely caught up in it. (FS) (-)

8. I try to look at everybody’s side of a disagreement before I make a decision. (PT)

9. When I see someone being taken advantage of, I feel kind of protective towards them. (EC)

10. I sometimes feel helpless when I am in the middle of a very emotional situation. (PD)

11. I sometimes try to understand my friends better by imagining how things look from their perspective. (PT)

12. Becoming extremely involved in a good book or movie is somewhat rare for me. (FS) (-)

13. When I see someone get hurt, I tend to remain calm. (PD) (-)

14. Other people’s misfortunes do not usually disturb me a great deal. (EC) (-)

15. If I’m sure I’m right about something, I don’t waste much time listening to other people’s arguments. (PT) (-)

16. After seeing a play or movie, I have felt as though I were one of the characters. (FS) 17. Being in a tense emotional situation scares me. (PD)

18. When I see someone being treated unfairly, I sometimes don’t feel very much pity for them. (EC) (-)

19. I am usually pretty effective in dealing with emergencies. (PD) (-)

20. I am often quite touched by things that I see happen. (EC)

21. I believe that there are two sides to every question and try to look at them both. (PT)

22. I would describe myself as a pretty soft-hearted person. (EC)

23. When I watch a good movie, I can very easily put myself in the place of a leading character. (FS)

24. I tend to lose control during emergencies. (PD)
25. When I’m upset at someone, I usually try to “put myself in his shoes” for a while. (PT)

26. When I am reading an interesting story or novel, I imagine how I would feel if the events in the story were happening to me. (FS)

27. When I see someone who badly needs help in an emergency, I go to pieces. (PD)

28. Before criticizing somebody, I try to imagine how I would feel if I were in their place. (PT)

Appendix B.

**KAMMUS (Kama Muta Multiplex Scale)**

Instructions:

Please answer the following questions about the video you just watched.

Or

Please answer the following questions about the event you just described.

{In all sections, Likert scales, 0 = “not at all” to 6 = “a lot”.)

*Section 1*

Please indicate whether you experienced any of the following sensations, feelings, or actions, and if so, to what extent:

1. Moist eyes.
2. Tears.
3. Goosebumps or hair standing up.
4. Chills or shivers.
5. A warm feeling in the center of the chest.
6. Some feeling in the center of the chest.
7. Choked up.
8. A lump in the throat.
9. Difficulty speaking.
10. I put one or both hands to my chest.
11. I took a deep breath or held my breath.
12. I said something like “awww.”
13. I smiled.
14. I giggled.
15. I frowned.
16. I lowered my head.

*Section 2*

Immediately *afterwards,* I felt

1. Buoyant or light.
2. Refreshed, energized, or exhilarated.

*Section 3*

Use the personal version for experiences in which you were directly involved.
Use the witnessing version for experiences that you observed, heard, or read about.
Fill out just one version, personal or witnessing, but not both.

**Participated (P)**

Please rate to what extent each of the following statements are true: ,

1. I felt an incredible bond.
2. I felt a special sense of belonging.
3. I felt an exceptional sense of closeness appear.
4. I felt the emergence of a remarkable feeling of oneness.
5. I felt a unique kind of love spring up.
6. I felt a phenomenal feeling of appreciating someone or being appreciated.
7. I felt an astonishing sense of needing a particular person or being needed.
8. I felt an extraordinary feeling of welcoming or being welcomed.
9. I felt that I gave or received exceptional care.
10. I felt that I gave or received a great kindness.
11. I felt exceptionally altruistic or someone was exceptionally altruistic toward me.
12. I felt that a connection between us was broken.
13. I felt that either I or someone else was comical.

**Witnessed (W)**

Please rate to what extent each of the following statements are true.

I observed, heard or read about…

Instruction for rating videos (Kaviar project and similar):
Now we would like you to answer the following questions regarding the video. Please indicate to what extent the following statements apply. While watching the video, I observed…

1. …an incredible bond.
2. …a special sense of belonging.
3. …an exceptional sense of closeness appear.
4. …the emergence of a remarkable feeling of oneness.
5. …a unique kind of love spring up.
6. …a phenomenal feeling of appreciating or being appreciated.
7. …an astonishing sense of someone needing a particular person or being needed by someone.
8. …an extraordinary feeling of welcoming or being welcomed.
9. …exceptional care being given to someone.
10. …a great kindness.
11. …an exceptional altruistic act.
12. …a connection that was broken.
13. …something comical.

*Section 4*

Please indicate whether you had each of the following feelings just afterwards, and if so, to what extent:

1. I felt like telling someone how much I care about them.
2. I wanted to hug someone.
3. I wanted to do something extra-nice for someone.
4. I felt especially friendly.
5. I felt more strongly committed to a relationship.
6. I wanted to be consoled.
7. I felt like joking.

*Section 5*

Please indicate whether each of the following was true, and if so, to what extent:

1. I was eager to tell my friends or family about the experience.
2. I wanted to have the experience together *with* others.

*Section 6*

Please indicate how positive you felt, and how negative you felt – you might have felt both positive *and* negative, one or the other, or neither.

1. I had positive feelings.
2. I had negative feelings.

*Section 7*

Please indicate whether each of the following was true, and if so, to what extent:

1. It was heartwarming.
2. I was moved.
3. I was touched.
4. It was a nostalgic moment.
5. It was a poignant experience.
6. I felt a part of something larger than myself.
7. I fell in love.
8. I felt sad.
9. I was awed.
10. I was amused.

Appendix C.

**Overview of Stimuli**

Most stimuli employed in the 16 studies are available in the figshare archive^1^. Links to the other stimuli are provided below the table. Note that the videos *Kitten2* and *Orphans edited* were deleted on Youtube and cannot be reproduced.

| Study # | Stimuli Type | Target Emotion | Stimuli Manipulation | Stimuli^1^ |
| --- | --- | --- | --- | --- |
| 1 | Narrative | Feeling Moved | n.a. | Study 1 Narratives |
| 2 | Video | Feeling Moved | Manipulation based on Communal Sharing conformations | Thai Medicine; Christian the Lion [Edited Version^2^]; Orphans |
| 3 | Video | Feeling Moved | n.a. | Thai Medicine; Math test; Dad |
| 4 | Video | Feeling Moved | n.a. | Marriage^3^; Human being^4^; Thai altruism^5^, Dad surprise^6^, Disabled surprise^7^; Sister^8^ |
| 5 | Video | Feeling Moved | Manipulated the intensity of intensification | Dog Homecoming; Soldier Homecoming |
| 6 | Video | Feeling Moved | Manipulated the identification with the protagonist | Dog Homecoming |
| 7 | Video | Feeling Moved, Neutral | n.a. | Bunny^9^; Kitten^10^; Kitten2; Kitten3^11^[Feeling Moved]/ Octopus^12^; Monkey^13^; Anglerfish^14^; Shark^15^ [Neutral] |
| 8 | Self-written narrative | Feeling Moved | n.a. | Valentines Day Tears Narratives |
| 9 | Self-written narrative | Feeling Moved | n.a. | Valentines Day Episodes Narratives |
| 10 | Audio narrative | Feeling Moved, Amusement, Neutral |  | Study10_Moving_Narrative/ Study10_Amusing_Narrative/Study10_Neutral_Narrative |
| 11 | Self-written narrative | Feeling Moved, Amusement, Neutral | n.a. | Mother Narratives |

Table continued

| Study # | Stimuli Type | Target Emotion | Stimuli Manipulation | Stimuli^1^ |
| --- | --- | --- | --- | --- |
| 12 | Video | Feeling Moved, Fear, Sadness, Happiness | n.a. | Thai Medicine [Moved]; The Shining [Fear]; Orphans [Sad]; Singing in the Rain [Happy] |
| 13 | Self-written narrative | Feeling Moved, Sadness | n.a. | Narratives Positive Negative Tears |
| 14 | Video | Feeling Moved | n.a. | Thai Medicine, Olympics, Oprah, Proposal, Talent; Orphans, Thai Altruism^5^, Reunion^16^ |
| 15 | Video | Feeling Moved | n.a. | Kitten4^17^, Kitten5^18^, Kitten6^19^, Kitten7^20^; Puppy^21^; Puppy2^22^; Puppy3^23^; Puppy4^24^ |
| 16 | Video | Feeling Moved, Sadness, Amusement, Awe | n.a. | Thai Medicine, Edeka^25^, Up^26^ [Moved]; Schindler’s List^27^, Life and Death^28^, Feed the World^29^ [Sadness]; Mary Dog^30^, Mary Gel^31^, Naked Gun^32^ [Amusement]; Dubai^33^, Dawn Wall^34^, BMX^35^ [Awe] |

^1^ <https://figshare.com/s/1f5affeac81983366205>

^2^ https://vimeo.com/98119720

^3^ <https://www.youtube.com/watch?v=s97tLze3pes>

^4^ <https://www.youtube.com/watch?v=VXyr0kAgrVU>

^5^ <https://www.youtube.com/watch?v=uaWA2GbcnJU>

^6^ <https://www.youtube.com/watch?v=yoV4TWiXI0k>

^7^ <https://www.youtube.com/watch?v=8hU6gR4zHnM>

^8^ <https://www.youtube.com/watch?v=Rfj4s7OaMZQ>

^9^ <https://www.youtube.com/watch?v=_v94XqFW4Qw>

^10^ <https://www.youtube.com/watch?v=JlWlnBWVQLE>

^11^ <https://www.youtube.com/watch?v=8HVWitAW-Qg>

^12^ <https://www.youtube.com/watch?v=H8oQBYw6xxc>

^13^ <https://www.youtube.com/watch?v=c1C9rM76BpI>

^14^ <https://www.youtube.com/watch?v=VqPMP9X-89o>

^15^ <https://www.youtube.com/watch?v=jZuUGJRtreI>

^16^ <https://www.youtube.com/watch?v=gHGDN9-oFJE&t=69s>

^17^ <https://www.youtube.com/watch?v=_RCqVy3TPkw>

^18^ <https://www.youtube.com/watch?v=ngxg-ZhojVY>

^19^ <https://www.youtube.com/watch?v=3w_gVmSEI2w>

^20^ <https://www.youtube.com/watch?v=qbnYWZKwgMk>

^21^ <https://www.youtube.com/watch?v=JfXMMoVQHHs>

^22^ <https://www.youtube.com/watch?v=gvJqMQy8P2M>

^23^ <https://www.youtube.com/watch?v=pBUxVELko10>

^24^ https://www.youtube.com/watch?v=zeln7wS5wPY

^25^ <https://youtu.be/EBDdM1HELvQ>

^26^ <https://youtu.be/A4VDGwzNQeY>

^27^ <https://youtu.be/Du_ZK92xWu8>

^28^ <https://youtu.be/2hTfTjAsuxk>

^29^ https://youtu.be/tWZr6eQLR4A

^30^ <https://youtu.be/EBG1uxttqis>

^31^ <https://youtu.be/gdJBPVjV7_w>

^32^ <https://youtu.be/j4IRoTAsIzk>

^33^ <https://youtu.be/QYKZwGi5Krk>

^34^ <https://youtu.be/fqxZA3ef8cI>

^35^ <https://www.youtube.com/watch?v=GOewG8nem4o>
